# Comparison of fecal microbiota and metabolites in diarrheal piglets pre-and post-weaning

**DOI:** 10.3389/fvets.2025.1613054

**Published:** 2025-06-20

**Authors:** Tao Huang, Jiangpeng Dong, Wenyu Zhang, Zhengyu Hu, Xuhui Tan, Hao Li, Kailing Sun, Ayong Zhao, Min Huang

**Affiliations:** Key Laboratory of Applied Technology on Green-Eco-Healthy Animal Husbandry of Zhejiang Province, College of Animal Science and Technology, College of Veterinary Medicine, Zhejiang A&F University, Hangzhou, China

**Keywords:** piglet, weaning, diarrhea, fecal microbiota, fecal metabolites

## Abstract

**Introduction:**

Diarrhea after weaning of piglets is one of the most serious diseases in pig herds, which brings huge economic losses to the pig industry. Diarrhea in piglets after weaning has a significant impact on the composition of gut microbiota, which leads to the disturbance of normal metabolic processes.

**Method:**

To investigate the effects of weaning diarrhea on gut microbiota and its metabolites, 84 samples were collected from 42 piglets pre-weaning and post-weaning, including 17 samples from post-weaning diarrheal piglets. The samples were sequenced for 16S rRNA and untargeted metabolites.

**Results:**

A total of 3,192 ASVs, 20 phyla, and 286 genera were identified. A total of 32 genera were detected by difference analysis between healthy post-weaning piglets and diarrhea post-weaning piglets. It was observed that post-weaning diarrhea led to a reduction in the relative abundance of *Bacteroides*, *UCG-002*, *Christensenellaceae_R-7_group*, *Escherichia-Shigella*, *Pyramidobacter*, *Lactobacillus* and a substantial increase in the relative abundance of *Prevotellaceae_NK3B31_group*, *Muribaculaceae*, *Prevotella*, *Holdemanella*, *Collinsella*, *Blautia*. A total of 360 differential metabolites were detected between healthy and diarrheal weaned piglets. 5-Hydroxyferulic acid methyl ester, D-Saccharic acid 1,4-lactone, 3-Methylhistidine were significantly enriched in post-weaning diarrheal piglets. These differential metabolites were enriched in the Nucleotide metabolism, ABC transporters, Biosynthesis of amino acids, Nicotinate and nicotinamide metabolism, Valine, leucine and isoleucine biosynthesis, Pyrimidine metabolism, Alanine, aspartate and glutamate metabolism, Purine metabolism, Caffeine metabolism, Butanoate metabolism pathways.

**Discussion:**

Taken together, this study systematically reveals the dynamic succession and structure of the gut microbiota and metabolites in diarrhea and healthy piglets pre-weaning and post-weaning. Our findings provide a reference for the microbiological and metabolite etiology associated with post-weaning diarrhea.

## 1 Introduction

Piglet diarrhea is a highly significant disease in pigs, having resulted in considerable economic losses for the pig industry (1, 2). To improve production efficiency, piglets are generally weaned within 3–4 weeks of birth, however, weaning stress causes diarrhea in piglets (3, 4). This stress disrupts gastrointestinal structure and function, causing villus atrophy, increased crypt depth, and alterations in gut microbiota structure (4, 5). Critically, these microbiota changes are both a consequence and contributor to diarrhea. Reduced microbial diversity is strongly associated with a heightened risk of post-weaning diarrhea and enteritis (6, 7). Weaning-induced impairment of the gut mucosal barrier allows pathogenic microorganisms to invade the gut, further destabilizing the microbiota (8).

The gut microbiota plays a key role in regulating nutrient metabolism in the gut, as well as contributing significantly to the establishment of an intact intestinal barrier and the development of a robust immune system within the gut (3). Disruption of this microbial balance impairs immunity and increases susceptibility to pathogens, compromising piglet physiology and even leading to death (9). The etiology of diarrhea of weaned piglets is multi-factorial. Weaning stress is the main pathogenic factor, and microbial infection is a cofactor. Recent studies have shown that weaning-related diarrhea in piglets leads to reduced diversity and structural changes in the gut microbiota, regardless of the underlying cause (2, 10, 11).

Metabolites are indispensable components that reflect biological activities and physiological functions. The complex relationship between the gut microbiota of piglets and the host can lead to the production of various metabolites. The co-existence of metabolites and gut microbiota constitutes the basic environment of the piglet gut. Intestinal flora and its metabolites have been shown to be key factors in the etiology of piglet diarrhea (10). Enterotoxigenic *Escherichia coli* adheres to the intestinal mucosa to produce heat-stable toxins and heat-unstable toxins, altering the water and electrolyte fluxes in the small intestine, thereby causing diarrhea (12–14). *Clostridium perfringens* type C binds to the small intestinal mucosal endothelial cells of piglets to produce beta toxin (CPB), which causes endothelial cell damage and diarrhea (12, 15).

The weaning process of piglets can cause changes in the composition and structure of the gut microbiota, and diarrhea after weaning further alters the gut microbiota (11, 16, 17). To observe the changes in gut microbiota and metabolites in piglets before and after weaning, as well as the effects of post-weaning diarrhea on gut microbiota and metabolites, 16S rRNA and non-targeted metabolome sequencing were performed on 42 Dongliao black piglets. In addition, we investigated the relationship between gut microbiota and their metabolites in weaned piglets with diarrhea. The results of this study provide a new theoretical basis for the development of new methods for preventing and controlling diarrhea in weaned piglets.

## 2 Materials and methods

### 2.1 Animal experimental design and sample collection

**Figure 1:**
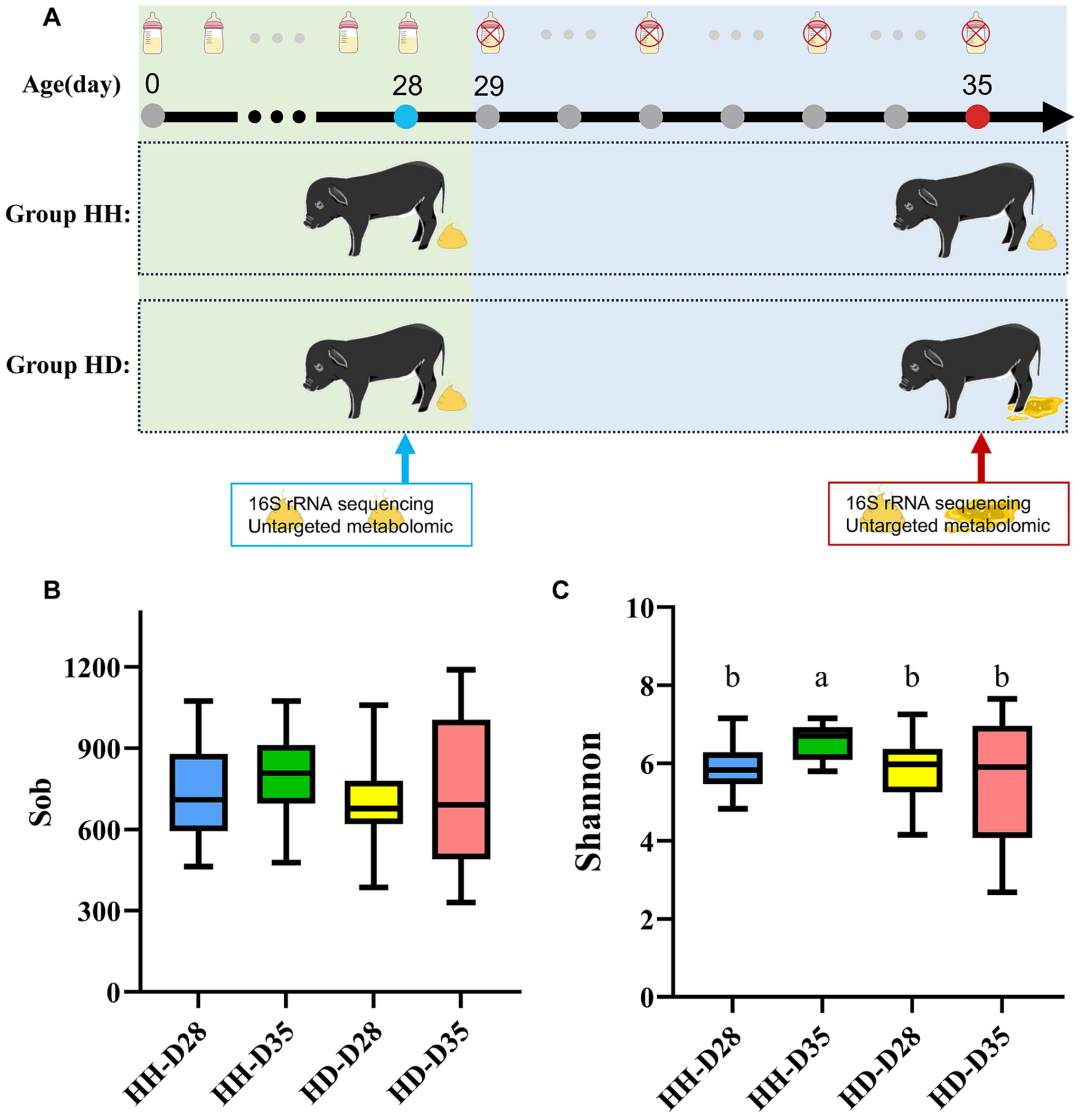
Experimental design and alpha diversity. **(A)** Sample collection time and grouping. **(B)** Sob index. **(C)** Shannon index.

Forty-two newborn piglets of Dongliao Black pig with similar body weight and body size were selected as experimental animals. The Dongliao Black pig is a breed that has been developed by selective breeding between the Chinese indigenous Min pig and the Berkshire pig. All 42 pigs in this experiment were raised on the Dongliao Black pig breeding farm (Shaoxing, Zhejiang, China). No antibiotics were used to treat any of the piglets. All piglets were raised by sows after birth and weaned at 28 days of age. After weaning, the piglets were separated from the sows and fed with commercial piglet-specific feed. All piglets did not show any diarrhea symptoms during the 28 days before weaning. Fresh fecal samples were collected on the morning of weaning. There were 17 piglets whose feces were watery or liquid-like for six consecutive days and these piglets were considered to have diarrhea. These 17 piglets were classified into the HD (Healthy at 28 days of age, diarrhea at 35 days of age) group. The other 25 piglets remained healthy without experiencing diarrhea or other diseases and were classified as the HH (Healthy at 28 days of age, Healthy at 35 days of age) group. Fresh feces were collected from piglets in the HH and HD groups at 35 days of age, and the samples were labeled as HH-D35 and HD-D35, respectively (Figure 1A). All fecal samples were collected in a 2 mL sterile freezer-tube, quickly frozen in liquid nitrogen, and subsequently stored in a refrigerator at –80°C until DNA and metabolites were extracted.

### 2.2 DNA extraction and 16S rRNA gene amplicon sequencing

DNA was extracted using QIAamp DNA Stool Mini Kit (Qiagen, Hilden, Germany) following the manual. Concentration and quality of the genomic DNA were checked by NanoDrop 2000 spectrophotometer (Thermo Fisher Scientific, Waltham, MA, United States). The V3-V4 hypervariable region of bacterial 16S rRNA gene was amplified with the universal primer 338F (5’-ACTCCTACGGGAGGCAGCAG-3′) and 806R (5’-GGACTACHVGGGTWTCTAAT-3′). The PCR was carried out on an ABI 9700 PCR instrument (Thermo Fisher Scientific, Waltham, MA, United States) using 25 μL reaction volumes, 3 μL BSA (2 ng/μL), 1 μL forward primer (5 μM), 1 μL reverse primer (5 μM), 2 μL template DNA, 12.5 μL 2 × Taq plus master mix and 5.5 μL ddH2O. Cycling parameters were 95°C for 5 min, followed by 28 cycles of 95°C for 45 s, 55°C for 50 s and 72°C for 45 s with a final extension at 72°C for 10 min (18). The PCR products were purified using an Agencourt AMPure XP Kit (Beckman Coulter, Inc., CA, United States). Sequencing libraries were generated with the NEB Next Ultra II DNA Library Preparation Kit (New England Biolabs, Inc., MA, United States) according to the manufacturer’s instructions. The library quality was assessed by Nanodrop 2000 (Thermo Fisher Scientific, Inc., MA, United States), Agilent 2,100 Bioanalyzer (Agilent Technologies, Inc., CA, United States), and ABI StepOnePlus Real Time PCR System (Applied Biosystems, Inc., CA, United States), successively. The purified amplicon was sequenced on the Illumina MiSeq sequencing platform (Illumina, CA, United States) according to the standard protocol of Majorbio bio-pharma Technology Co. Ltd. (Shanghai, China).

### 2.3 16S rRNA sequence data analysis

The use of Pear (v0.9.6) software is recommended for the filtering and splicing of raw data (19). Sequences were excluded from further consideration if they contained ambiguous bases (N) and if the sequence quality score was below 20. During the splicing process, the minimum overlap setting was 10 bp. Following the splicing process, the Vsearch (v2.7.1) software was employed to remove sequences with a length of less than 230 bp (20). Additionally, chimeric sequences were removed using the UCHIME method in accordance with the Gold Database. Sequences were then clustered into amplified sequence variants (ASVs) (21). The data were optimized using DADA2 to obtain representative ASV sequences and abundance information (22). All ASV representative sequences were annotated to the Silva138 Database (23). The alpha-diversity index and beta-diversity of microbiota based on Bray-Curtis distances were performed in QIIME2 (24). The Net Relatedness Index (NRI) and Nearest Taxon Index (NTI) were calculated using picante package of R. Principal coordinate analysis (PCoA) of the Bray-Curtis distances between samples was performed, and the clustering between samples was evaluated using the permutational multivariate analysis of variance (PERMANOVA) with the vegan package (v.2.6–4) in R (25). Random-forest models (ntree = 1,000) were constructed using the randomForest package in R to determine the bacterial genera and metabolites that could be used to distinguish between diarrheal and healthy piglets. A 10-fold cross-validation procedure was conducted using the rfcv function of the randomForest package. The interpolated area under the receiver operating characteristic (ROC) curve (AUC) was determined using the pROC3 package of R to evaluate the diagnostic accuracy of the model. Inference of microbiota functional profiles was performed with PICRUSt2 (v2.2.0) (26).

### 2.4 Untargeted metabolome profiling of feces samples

Twenty-five milligrams of the fecal sample was weighed to an EP tube, mixed with 500 μL extract solution (methanol: acetonitrile: water = 2: 2: 1). The samples were then homogenized at 35 Hz for 4 min and sonicated in an ice water bath for 5 min. The homogenisation and sonication cycle was repeated 3 times. The sample was then centrifuged at 12,000 rpm (RCF = 13,800 (×g), R = 8.6 cm) for 15 min at 4°C. The supernatant obtained in this way was transferred to a fresh glass vial for the LC/MS analysis. An equal aliquot of the supernatant from each sample was mixed to prepare the quality control sample. The analyses were carried out using a UHPLC (Thermo Fisher Scientific, Waltham, MA, United States) coupled to an Orbitrap Exploris 120 mass spectrometer (Thermo Fisher Scientific, Waltham, MA, United States).

The raw data were converted to the mzXML format using ProteoWizard in the process of data analysis. As a first step, metabolite features detected in more than 50% of the experimental samples were retained for further analysis. Subsequently, missing values in the original data were imputed using half of the minimum value. Furthermore, features with a relative standard deviation exceeding 30% were eliminated from subsequent analysis (27). Following these preprocessing steps, the X peak was identified and preserved along with its corresponding metabolite. The resulting peak number, sample name, and normalized peak area were then inputted into the R software package MetaboAnalystR for further analysis. Subsequently, an in-house program developed in R, based on XCMS, was employed for peak detection, extraction, alignment, and integration. Molecular mass data (m/z) were compared using HMDB database for metabolite annotation (28). The resulted data involving the peak number, sample name, and normalized peak area were fed to R package MetaboAnalystR for principal component analysis (PCA) and orthogonal partial least squares discriminant analysis (OPLS-DA) (29). To refine this analysis, the first principal component of variable importance in the projection (VIP) was obtained. The VIP values summarize the contribution of each variable to the model. The metabolites with VIP > 1, *p* < 0.05 were considered as significantly changed metabolites. In addition, commercial databases including KEGG^1^ and MetaboAnalyst^2^ were utilized to search for the pathways of metabolites.

### 2.5 Statistical analysis

Unless otherwise specified, all statistical tests were conducted in R (v 4.3.1). The Kruskal-Wallis test was used for the multiple group comparisons, and the Wilcoxon rank sum test with FDR correction was used to pairwise compare between groups. The spearman correlation analysis between significantly different gut microbials (genus level) and differential metabolites was performed using R (v 4.3.1). The differential metabolites were log_2_(Fold Change) > 5 between HD-D28 and HD-D35 for correlation analysis.

## 3 Results

### 3.1 16S rRNA gene sequencing data analysis

A total of 84 fecal samples from 42 piglets were collected at two different time points of 28 days and 35 days of age, and the V3-V4 hypervariable region of 16S RNA amplicon was sequenced. A total of 5,621,635 high-quality filtered reads were obtained, with an average of 66,924 sequences per sample. Based on these sequences, a total of 31,920 ASVs were identified. These ASVs were classified into microbial taxa and assigned to a total of 20 phyla or 286 genera. A summary of the sequencing data and the microbial taxa for each of the fecal samples can be found in Supplementary Table S1.

### 3.2 Microbial diversity of feces microbiota

To investigate the effects of weaning and diarrhea on the gut microbiota of piglets, the observed species richness index and the shannon index were used to calculate the alpha diversity of each group at the ASV level (Figures 1B,C). The difference analysis of observed species index showed that there was no significant difference among the four groups (Kruskal-Wallis test, *p* > 0.05). No significant differences were identified between samples from the pre-and post-weaning periods in both the healthy and diarrhea groups (Wilcoxon rank sum test, *p* > 0.05, Figure 1B). There were significant differences in the shannon index of feces microbiota among the four groups (Kruskal-Wallis test, *p* = 3.11 × 10^−3^). The shannon index of HH samples was significantly different between pre-and post-weaning periods (*p* = 2.54 × 10^−4^). There was significant difference in shannon index between healthy (HH-D35) and diarrheal piglets (HD-D35) on 35 days of age (*p* = 0.03, Figure 1C). The NRI of the HH-D35 group was significantly higher than that of the HD-D35 group (*p* = 4.36 × 10^−3^, Supplementary Figure S2A). Correspondingly, the NTI also exhibited the same pattern as the NRI (*p* = 5.20 × 10^−3^, Supplementary Figure S2B).

**Figure 2:**
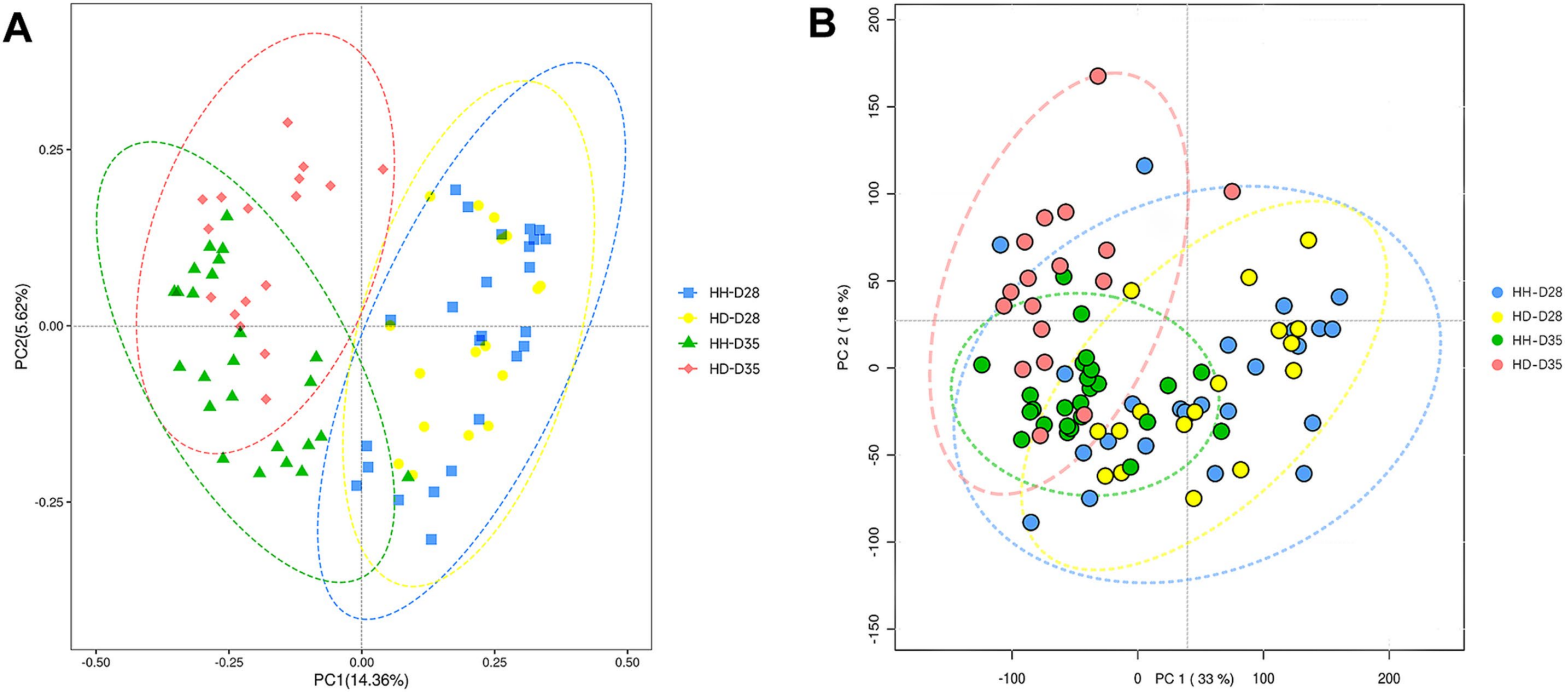
Structure of fecal microbiota and metabolites. **(A)** Principal coordinates analysis (PCoA) of fecal microbial communities. **(B)** Principal component analysis (PCA) of fecal metabolomics.

To assess the extent of similarity in the overall structure of the gut microbiota, we performed a PCoA at the ASV level using Bray-Curtis distances (Figure 2A). Samples were significantly clustered according to pre-weaning and post-weaning (PERMANOVA, *p* = 3.00 × 10^−3^). The samples from HH-D28 and HH-D35 groups clustered separately (PERMANOVA, *p* = 6.10 × 10^−3^) (Supplementary Figure S3A). Most of the fecal samples from the HD-D35 group and the HD-D28 group clustered dispersedly, but there was some overlap (PERMANOVA, *p* = 0.034) (Supplementary Figure S3B). The HH-D28 and HD-D28 samples clustered closely (PERMANOVA, *p* = 0.515). After weaning, diarrhea samples (HD-D35) were clustered with partial overlap to the healthy samples (HH-D35) (PERMANOVA, *p* < 0.001).

### 3.3 Composition and differences of microbiota

**Figure 3:**
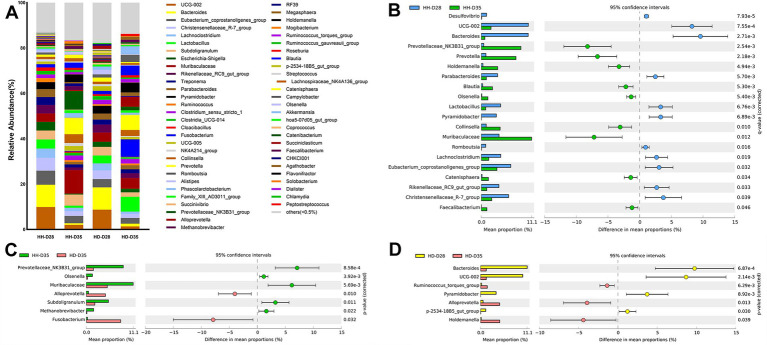
Comparison and differences of fecal microbiota in piglets. **(A)** Composition of genera in different groups. **(B)** Significantly different genera between HH-D28 and HH-D35 groups. Only the top 20 significantly different genera with the highest relative abundance were shown. **(C)** Significantly different genera between HH-D35 and HD-D35 groups. **(D)** Significantly different genera between HD-D28 and HD-D35 groups. The mean relative abundance of genera differences between the two groups > 1% were shown. Welch’s *t*-test with Benjamini-Hochberg FDR correction was used to test for significant differences (*p* < 0.05) between groups.

A total of 20 phyla were detected in all samples, among which Firmicutes (55.96%) and Bacteroidota (27.14%) were the most enriched, followed by Actinobacteriota (3.31%), Proteobacteria (2.85%), and Synergistota (2.72%) (Supplementary Figure S1). At the genus level, a total of 286 genera were detected, among which an overview of the relevant abundance of >0.5% is represented in Figure 3A. The most dominant genera in all the samples were *Muribaculaceae* (5.99%), *UCG-002* (5.56%), *Bacteroides* (5.40%), *Prevotella* (4.16%), *Eubacterium_coprostanoligenes_group* (4.04%), *Subdoligranulum* (3.79%), *Lactobacillus* (3.49%), *Christensenellaceae_R-7_group* (3.44%), *Prevotellaceae_NK3B31_group* (3.03%), and *Rikenellaceae_RC9_gut_group* (2.57%) (Figure 3A and Supplementary Table S2). In the samples of the HH-D28 group, the most predominant genera were *UCG-002* (9.86%), *Bacteroides* (9.86%), *Eubacterium_coprostanoligenes_group* (6.17%), *Christensenellaceae_R-7_group* (5.65%), *Lachnoclostridium* (4.08%), *Lactobacillus* (4.03%), *Subdoligranulum* (3.91%), *Escherichia-Shigella* (3.84%), *Muribaculaceae* (3.75%), and *Rikenellaceae_RC9_gut_group* (3.68%). As the samples of the HD-D28 group were also collected from healthy piglets before weaning, the composition of the genera was very similar to that of HH-D28, and the most enriched genera were *Bacteroides* (9.82%), *UCG-002* (8.71%), *Muribaculaceae* (4.11%), *Christensenellaceae_R-7_group* (3.82%), *Subdoligranulum* (3.81%), *Eubacterium_coprostanoligenes_group* (3.67%), *Rikenellaceae_RC9_gut_group* (3.48%), *Lactobacillus* (3.44%), *Pyramidobacter* (3.41%), *Lachnoclostridium* (3.12%), and *Parabacteroides* (2.66%). After weaning, the composition of genera in healthy piglets (HH-D35) changed and the most predominant genera were *Muribaculaceae* (10.37%), *Prevotellaceae_NK3B31_group* (8.2%), *Prevotella* (7.22%), *Subdoligranulum* (4.9%), *Collinsella* (3.9%), *Holdemanella* (3.43%), *Eubacterium_coprostanoligenes_group* (3.2%), *Blautia* (2.34%), *UCG-005* (2.22%), and *Christensenellaceae_R-7_group* (2.08%). The most enriched genera of piglets with post-weaning diarrhea (HD-D35) were *Fusobacterium* (7.72%), *Prevotella* (6.68%), *Lactobacillus* (6.54%), *Muribaculaceae* (4.73%), *Holdemanella* (4.39%), *Alloprevotella* (4.28%), *Blautia* (4.24%), *Akkermansia* (3.49%), *Campylobacter* (3.14%), and *Collinsella* (2.61%).

To compare the effects of weaning and diarrhea on genera, we performed differential analyses of bacterial genera between different groups of samples. Genera with average relative abundance < 0.05% were filtered out, resulting in 119 genera for further analysis. Comparing the differences in genera of healthy piglets between HH-D28 (pre-weaning) and HH-D35 (post-weaning), a total of 38 bacterial genera were detected with significant differences. The relative abundance of bacterial genera *Bacteroides*, *UCG-002*, *Christensenellaceae_R-7_group*, *Escherichia-Shigella*, *Pyramidobacter*, *Lactobacillus*, *Eubacterium_coprostanoligenes_group*, *Rikenellaceae_RC9_gut_group*, *Lachnoclostridium*, *Parabacteroides*, *Romboutsia* were significantly higher in samples of HH-D28 than in samples of HH-D35. In contrast, the relative abundances of bacterial genera *Prevotellaceae_NK3B31_group*, *Muribaculaceae*, *Prevotella*, *Holdemanella*, *Collinsella*, *Blautia*, *Catenisphaera*, *Olsenella*, *Faecalibacterium* were significantly higher in HH-D35 samples compared to HH-D28 samples (Figure 3B). To compare the effects of post-weaning diarrhea on genera, a total of 32 genera were detected by difference analysis between HH-D35 and HD-D35 samples. The relative abundances of *Fusobacterium*, *Alloprevotella*, *T34* were significantly higher in HD-D35 (diarrhea) than those in HH-D35 (healthy), while the relative abundances of genera *Prevotellaceae_NK3B31_group*, *Muribaculaceae*, *Subdoligranulum*, *Methanobrevibacter*, *Olsenella*, *Colidextribacter*, *Eubacterium_siraeum_group* were significantly higher in HH-D35 samples (Figure 3C). There were 15 genera that had significant differences between HD-D28 and HD-D35 samples, among which *Bacteroides*, *UCG-002*, *Pyramidobacter*, *p-2534-18B5_gut_group*, *Desulfovibrio*, *Cloacibacillus*, *Butyricimonas* were significantly higher in the HD-D28, while *Holdemanella*, *Alloprevotella*, *Collinsella*, *Ruminococcus_torques_group*, *Dorea*, *Lachnospiraceae_NK4A136_group*, *T34*, *UCG-008* were significantly higher in the HD-D35 (Figure 3D).

Random forest analysis identified 17 bacterial genera that can be used to distinguish between healthy and diarrheic piglet samples, with a diagnostic accuracy of the AUC as high as 93.94% and a 95% confidence interval of 92.91–100%. These genera biomarkers were *Clostridium_sensu_stricto_6*, *Ruminococcus*, *Odoribacter*, *Pseudoflavonifractor*, *Oscillibacter*, *Phascolarctobacterium*, *Incertae_Sedis*, *Lactobacillus*, *UBA1819*, *Oscillospira*, *Colidextribacter*, *Clostridia_UCG_014*, *Methanobrevibacter*, *Prevotellaceae_NK3B31_group*, *Slackia*, *Streptococcus*, and *Alloprevotella* (Supplementary Figures S4A,C).

### 3.4 Predicted functional capacities of microbiota

Predict Kyoto Encyclopedia of Genes and Genomes (KEGG) and KEGG Orthologies (KOs) using 16S rRNA sequencing data to investigate functional differences in piglet microbial communities induced by weaning and diarrhea. A total of 167 KOs were identified in all samples. The results of the study demonstrated that a total of 81 KOs exhibited significant differences in post-weaning healthy piglets (Supplementary Table S3). Forty KOs were more enriched in pre-weaning healthy piglets (HH-D28), including drug metabolism-other enzymes, flagellar assembly, bacterial chemotaxis, tetracycline biosynthesis, biotin metabolism, chloroalkane and chloroalkene degradation, polyketide sugar unit biosynthesis, lipoic acid metabolism, synthesis and degradation of ketone bodies, citrate cycle (TCA cycle). While 41 KOs showed significantly higher abundance in post-weaning healthy piglets (HH-D35), most of them were correlated with the biosynthesis of ansamycins, biosynthesis of vancomycin group antibiotics, D-Glutamine and D-glutamate metabolism, photosynthesis, phosphotransferase system (PTS), peptidoglycan biosynthesis, fructose and mannose metabolism, one carbon pool by folate, carbon fixation in photosynthetic organisms, aminoacyl-tRNA biosynthesis, etc.

After weaning (35 days of age), a total of 10 significant differences in KOs were detected between healthy (HH-D35) and diarrheal piglets (HD-D35). The HH-D35 healthy piglets exhibited a higher abundance of KOs associated with RNA polymerase, histidine metabolism, cysteine and methionine metabolism, peptidoglycan biosynthesis, terpenoid backbone biosynthesis, alanine, aspartate and glutamate metabolism, glycine, serine and threonine metabolism, protein processing in endoplasmic reticulum. In contrast, the HD-D35 diarrheal piglets showed more pronounced associations with glycerophospholipid metabolism (Supplementary Table S3).

Focusing on both weaning and diarrhea, a total of 13 KOs with significant differences were detected by comparing the microbial functions of healthy piglets before weaning (HD-D28) and those with diarrhea after weaning (HD-D35). In healthy piglets before weaning, the predominant enriched KOs were associated with drug metabolism-other enzymes, citrate cycle (TCA cycle), carbon fixation pathways in prokaryotes, valine, leucine and isoleucine degradation, glycine, serine and threonine metabolism, protein processing in endoplasmic reticulum. The enriched KOs in the diarrheal piglets after weaning were phosphotransferase system (PTS), galactose metabolism, fructose and mannose metabolism, starch and sucrose metabolism, neomycin, kanamycin and gentamicin biosynthesis, amino sugar and nucleotide sugar metabolism, glycerophospholipid metabolism (Supplementary Table S3).

### 3.5 Fecal metabolomic features in healthy and diarrheic piglets

**Figure 4:**
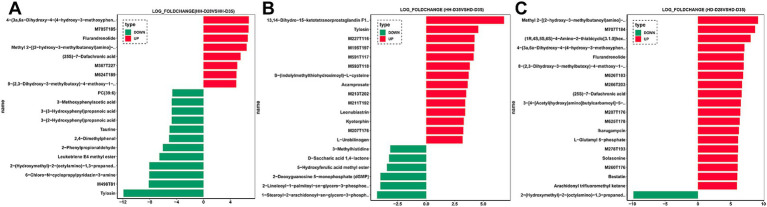
Differences in fecal metabolites of piglets. **(A)** HH-D28 vs. HH-D35. **(B)** HH-D35 vs. HD-D35. **(C)** HD-D28 vs. HD-D35. Only the differential metabolites with the top 20 log fold change values are shown.

**Figure 5:**
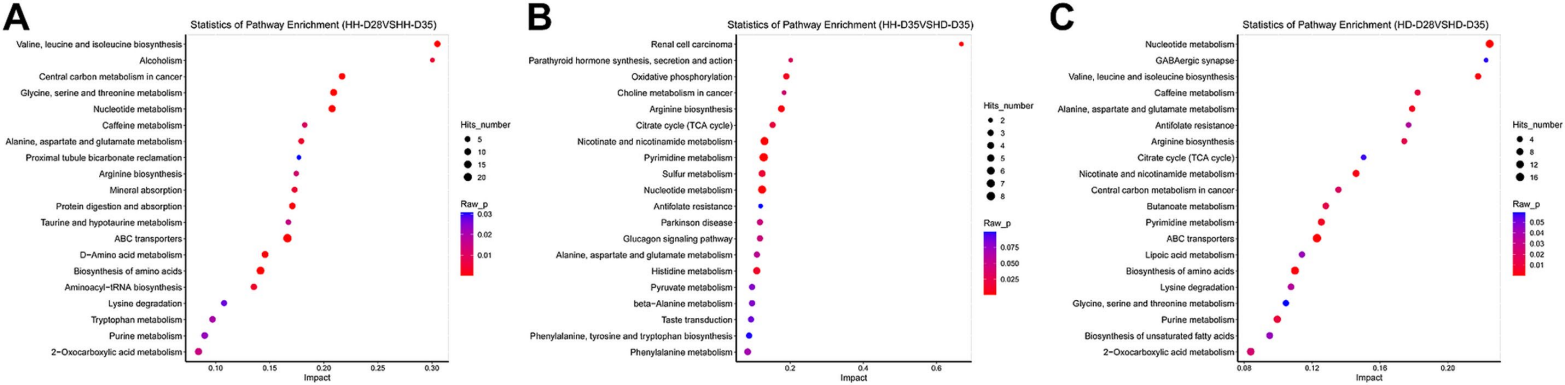
Pathway enrichment of differential metabolites. **(A)** HH-D28 vs. HH-D35. **(B)** HH-D35 vs. HD-D35. **(C)** HD-D28 vs. HD-D35.

A total of 84 fecal samples from healthy and diarrheal piglets were collected for untargeted metabolomics sequencing, and 1,182 metabolites were identified. PCA analysis showed that there were significant differences of overall metabolic abundance in different groups (Figure 2B, PERMANOVA, *p* < 0.05). To investigate the effect of weaning on fecal metabolites in piglets, we compared pre- (HH-D28) and post-weaning (HH-D35) fecal metabolite differences in healthy piglets, and found 487 significantly different metabolites. A total of 237 metabolites were found to be more abundant in pre-weaning healthy piglets (HH-D28), including 4-(3a,6a-Dihydroxy-4-(4-hydroxy-3-methoxyphenyl)tetrahydro-1H,3H-furo[3,4-c]furan-1-yl)-2-methoxyphenyl beta-D-glucopyranoside, M785T185, Flurandrenolide, Methyl 2-[(2-hydroxy-3-methylbutanoyl)amino]-3-methylbutanoate, (25S)-7-Dafachronic acid, M367T227, M624T189, 8-(2,3-Dihydroxy-3-methylbutoxy)-4-methoxy-1-methylquinolin-2(1H)-one, etc. In the post-weaning healthy piglets (HH-D35), 250 differential metabolites were detected with higher abundance, including Tylosin, M498T81, 6-Chloro-N-cyclopropylpyridazin-3-amine, 2-(Hydroxymethyl)-2-(octylamino)-1,3-propanediol, Leukotriene E4 methyl ester, 2-Phenylpropionaldehyde, 2,4-Dimethylphenol, Taurine, 3-(2-Hydroxyphenyl)propanoic acid, 3-Methoxyphenylacetic acid, 3-(3-Hydroxyphenyl)propanoic acid, PC(39:6), etc. (Figure 4A). To further investigate these differential metabolite functions, perform metabolic pathway analyses using the KEGG database. The results indicated a significant enrichment of metabolic pathways, including ABC transporters, Nucleotide metabolism, Biosynthesis of amino acids, Glycine, Serine and threonine metabolism, Valine, Leucine and isoleucine biosynthesis, Central carbon metabolism in cancer, D-Amino acid metabolism, Protein digestion and absorption, Aminoacyl-tRNA biosynthesis, Alanine, Aspartate and glutamate metabolism, Mineral absorption, Alcoholism, Caffeine metabolism, Arginine biosynthesis, 2-Oxocarboxylic acid metabolism, Taurine and hypotaurine metabolism, Tryptophan metabolism, Purine metabolism, Lysine degradation, Proximal tubule bicarbonate reclamation (Figure 5A).

To investigate the effects of post-weaning diarrhea on fecal metabolites, we compared the metabolites of HH-D35 and HD-D35 fecal samples. A total of 360 differential metabolites were detected between healthy and diarrheal weaned piglets, of which 254 differential metabolites in healthy piglets had a higher abundance, including 13,14-Dihydro-15-ketotetranorprostaglandin F1beta, Tylosin, M227T116, M195T197, M591T117, M593T118, S-(Indolylmethylthiohydroximoyl)-L-cysteine, M213T202, M211T192, Leonubiastrin, Kyotorphin, M207T176, L-Urobilinogen, and etc. In post-weaning diarrheal piglets, 1-Stearoyl-2-arachidonoyl-sn-glycero-3-phospho-(1-myo-inositol), 2-Linoleoyl-1-palmitoyl-sn-glycero-3-phosphoethanolamine, 2-Deoxyguanosine 5-monophosphate (dGMP), 5-Hydroxyferulic acid methyl ester, D-Saccharic acid 1,4-lactone, and 3-Methylhistidine were significantly enriched (Figure 4B). The differential metabolites were associated with Pyrimidine metabolism, Nicotinate and nicotinamide metabolism, Nucleotide metabolism, Renal cell carcinoma, Arginine biosynthesis, Oxidative phosphorylation, Histidine metabolism, Sulfur metabolism, Citrate cycle (TCA cycle), Parathyroid hormone synthesis, secretion and action (Figure 5B). Random forest analysis discovered 10 metabolites that can be utilized to distinguish between healthy and diarrheal piglet samples, with an AUC diagnostic accuracy of up to 95.71% and a 95% confidence range of 87.37 to 100%. Leu-Thr, Riboflavin, 6-Amino-4-hydroxy-2-naphthalenesulfonicacid, 6-Acetyl-D-glucose, Cytidine2, 3-cyclicphosphate, Nicotinamide, delta-Tocotrienol, N-(2-Hydroxy-3-methylbutanoyl)valine, Leonubiastrin, andM211T192 were the main metabolite biomarkers (Supplementary Figures S4B,D).

Focusing on both weaning and diarrhea, we compared metabolite differences between HD-D28 and HD-D35 fecal samples and found a total of 468 significantly different metabolites. In pre-weaning healthy piglets (HD-D28), 325 significantly different metabolites had higher abundances, among which Methyl 2-[(2-hydroxy-3-methylbutanoyl)amino]-3-methylbutanoate, M707T184, (1R,4S,5S,6S)-4-Amino-2-thiabicyclo[3.1.0]hexane-4,6-dicarboxylic acid 2,2-dioxide, 4-(3a,6a-Dihydroxy-4-(4-hydroxy-3-methoxyphenyl)tetrahydro-1H,3H-furo[3,4-c]furan-1-yl)-2-methoxyphenyl beta-D-glucopyranoside, Flurandrenolide, 8-(2,3-Dihydroxy-3-methylbutoxy)-4-methoxy-1-methylquinolin-2(1H)-one, M626T183, M266T203, (25S)-7-Dafachronic acid, and 3-[4-[Acetyl(hydroxy)amino]butylcarbamoyl]-5-[3-[acetyl(hydroxy)amino]propylamino]-3-hydroxy-5-oxopentanoic acid. In post-weaning diarrheal piglets, “2-(Hydroxymethyl)-2-(octylamino)-1,3-propanediol,” 6-Chloro-N-cyclopropylpyridazin-3-amine, 2-Phenylpropionaldehyde, 3-Pyridylacetic acid, Trigonelline, alpha-Linolenic acid, gamma-Linolenic acid, SM(d36:2), Maltose, and Melibiose were more abundant than in pre-weaning healthy piglets (HD-D28) (Figure 4C). These differential metabolites were enriched with the Nucleotide metabolism, ABC transporters, Biosynthesis of amino acids, Nicotinate and nicotinamide metabolism, Valine, leucine and isoleucine biosynthesis, Pyrimidine metabolism, Alanine, aspartate and glutamate metabolism, Purine metabolism, Caffeine metabolism, and Butanoate metabolism pathways (Figure 5C).

### 3.6 The association between fecal microbiota and metabolites in diarrheic and healthy piglets

**Figure 6:**
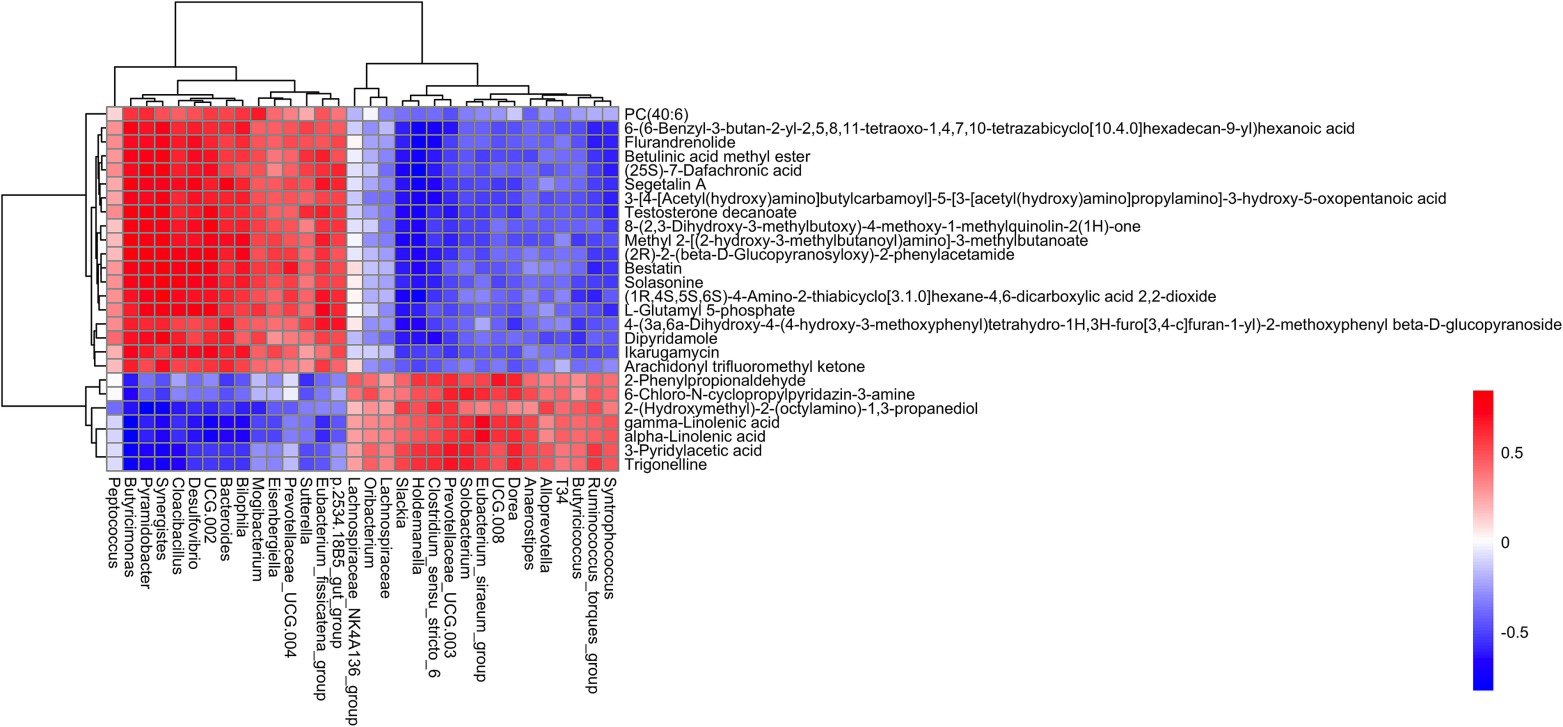
The correlations between fecal microbiota and metabolites.

Correlation analysis was performed between differential metabolites and microbiota significantly associated with weaning and diarrhea. There were significant positive correlations between 19 different metabolites and 15 bacterial genera, such as Flurandrenolide, Betulinic acid methyl ester, Segetalin A, Testosterone decanoate, Bestatin, Solasonine, L-Glutamyl 5-phosphate, Dipyridamole, and Ikarugamycin, exhibited positive correlation with *Bilophila*, *Bacteroides*, *UCG-002*, *Desulfovibrio*, *Cloacibacillus*, *Synergistes*, *Pyramidobacter*, and *Butyricimonas*. In contrast, these 19 metabolites were significantly negatively correlated with 17 bacterial genera, including *Syntrophococcus*, *Ruminococcus_torques_group*, *Butyricicoccus*, *Clostridium_sensu_stricto_6*, *Holdemanella*, *Slackia*, and etc. The 2-Phenylpropionaldehyde, 6-Chloro-N-cyclopropylpyridazin-3-amine, 2-(Hydroxymethyl)-2-(octylamino)-1,3-propanediol, gamma-Linolenic acid, alpha-Linolenic acid, 3-Pyridylacetic acid, and Trigonelline were significantly positively correlated with 17 genera, including *Syntrophococcus*, *Ruminococcus_torques_group*, *Butyricicoccus*, *T34*, *Alloprevotella*, *Anaerostipes*, *Dorea*, and etc. On the contrary, these metabolites were significantly negatively correlated with 17 bacteria genera such as *Bilophila*, *Bacteroides*, *UCG-002*, *Desulfovibrio*, *Cloacibacillus*, *Synergistes*, *Pyramidobacter*, *Butyricimonas*, and etc. (see Figure 6).

## 4 Discussion

Piglets are generally weaned within 3–4 weeks of birth to improve production efficiency (3). The weaning period is a critical milestone in piglet early life, during which the transition from breast milk to solid feed induces multifaceted gastrointestinal challenges. Weaning stress disrupts intestinal barrier function, triggers villus atrophy, and elevates pro-inflammatory factors (e.g., TNF-*α*, IL-6), collectively driving dysbiosis in gut microbiota composition (30). This microbial imbalance, in turn, exacerbates diarrhea, reduces feed intake, and impairs growth—a major contributor to economic losses in swine production (2, 11). Numerous previous studies have consistently shown that weaning-induced changes in nutrient absorption and intestinal physiology collectively reshape the gut microbiota’s distribution, composition, and metabolic activity (31, 32). These modifications highlight the microbiota’s central role in mediating the adverse effects of weaning stress, linking microbial dysregulation to both intestinal inflammation and impaired growth performance. The dietary shift exerts a significant impact on the succession of intestinal microbial communities in piglets (33, 34). This study found that there was no significant difference in the observed species index of gut microbiota in post-weaning piglets compared to pre-weaning, but there was a significant difference in the Shannon index, which is consistent with previous studies (4, 35, 36). Firmicutes and Bacteroides are the predominant bacteria with the highest relative abundance pre-and post-weaning, followed by Actinobacteria, Proteobacteria and Clostridium, which is consistent with previous research results (35, 37).

We observed that post-weaning diarrhea led to a reduction in the relative abundance of *Bacteroides* and a substantial increase in the relative abundance of *Clostridium* and *Campylobacter*. *Bacteroides* play a key role in maintaining intestinal homeostasis, establishing a robust intestinal barrier and ensuring normal growth and development as a major component of the early life intestinal microbial composition in pigs (38). *Campylobacter* is one of the most common causes of bacterial diarrhea, with studies suggesting that it is responsible for exclusive diarrhea in approximately 15% of piglets (39). We therefore hypothesize that the reduction in *Bacteroides* abundance and concomitant increase in *Campylobacter* abundance observed during the experiment may contribute to the occurrence of post-weaning diarrhea in piglets. We observed a significant decrease in the abundance of *Butyricimona*, *Bacteroides*, *UCG-002*, *Pyramidobacter* in the fecal samples of piglets from the post-weaning diarrhea samples. Conversely, *Ruminococcus_torques_group*, *Alloprevotella*, *T34*, *UCG-008*, *Dorea*, *Holdemanella* and *Collinsella* exhibited a significant increase. *Butyricimona* is considered a beneficial bacterium because of its ability to produce butyric acid and isobutyric acid, which play a crucial role in the host’s anti-inflammatory response and immune regulation (40, 41). Previous studies have demonstrated the critical role of *UCG-002* in modulating the gut microbiota. As a methanogenic bacterium, *UCG-002* actively participates in metabolizing various nutrients in the body, having a profound influence on maintaining normal intestinal function and overall physical well-being (42, 43). *Pyramidobacter* may affect the host immune system by regulating IGA (44). *Alloprevotella* was negatively correlated with the levels of IL-4 and IL-10, which are indicators of host immune inflammatory response factors (45). In early-weaned pigs, *Holdemanella* showed a significant positive correlation with volatile fatty acid (e.g., propionic acid) metabolism, and its increased abundance was linked to reduced diarrhea incidence (46).

Predicted KEGG and KO analysis for microbiota revealed distinct metabolic shifts associated with weaning and diarrhea in piglets. In post-weaning healthy piglets (HH-D35), the enriched KOs related to D-Glutamine and D-glutamate metabolism suggest enhanced microbial capacity for nitrogen metabolism, potentially aiding adaptation to solid diets (47). The upregulation of PTS (phosphotransferase system) and peptidoglycan biosynthesis in HH-D35 implies improved carbohydrate utilization and intestinal barrier maintenance, aligning with previous findings that dietary transition promotes microbial specialization in energy metabolism (48). Notably, the comparison of functional differences between healthy piglets (HH-D35) and diarrheal piglets (HD-D35) revealed that the fecal microbial function of diarrheal piglets dominated the glycerophospholipid metabolism, which may reflect intestinal inflammation or membrane lipid remodeling under dysbiosis, consistent with studies linking phospholipid metabolism to gut barrier dysfunction (49).

We investigated the influence of post-weaning diarrhea on the composition of fecal metabolites in piglets. It was found that the concentrations of metabolites such as Flurandrenolide, Ikarugamycin and Solasonine in piglets with post-weaning diarrhea were significantly reduced. Flurandrenolide plays a key role in the body’s immune response to inflammation (50). Ikarugamycin regulates the activity of T-cells in the body and is involved in a variety of immune responses (51). The alkaloid solasonine is actively involved in a number of sugar metabolism pathways in the intestine, effectively increasing the rate of utilization of polysaccharides in the gastro-intestinal tract and promoting optimal growth performance in animals (52). We found that the concentrations of gamma-Linolenic acid, alpha-Linolenic acid, Melibiose, N-Acetylputrescine and other metabolites increased significantly after diarrhea in piglets. N-Acetylputrescine concentration was significantly increased in patients with ulcerative colitis, suggesting that it is associated with intestinal inflammation (53).

KEGG enrichment analysis of differential metabolites revealed significant changes in pathways including GABAergic synapse, Antifolate resistance, Citrate cycle (TCA cycle), Lipoic acid metabolism, Lysine degradation, and Biosynthesis of unsaturated fatty acids after weaning. During the weaning period, the change of nutrient absorption of piglets has a profound influence on the energy conversion pathway in the body (54). Since piglets no longer absorb sugars and other nutrients directly from breast milk after weaning, but rely more on gut microbes for post-metabolic absorption of complex diets, there is a rapid shift in the structure of small molecule metabolites and flora involved in metabolism (55).

The correlation analysis between fecal microbiota and metabolites revealed complex interactions underlying weaning stress and diarrhea in piglets. The anti-inflammatory and immunomodulatory metabolites gamma-Linolenic acid, alpha-Linolenic acid, and Trigonelline showed positive associations with commensal bacteria (*Butyricicoccus*, *Anaerostipes*), which are known for reinforcing gut barrier function and producing butyrate (56–58). The gamma-linolenic acid and alpha-linolenic acid were significantly negatively correlated with *Desulfovibrio*. Gamma-linolenic acid and alpha-linolenic acid belong to polyunsaturated fatty acids and can inhibit the immune response mediated by NF-κB and MAPK signaling pathways by reducing the protein level of nitric oxide synthase (59). *Desulfovibrio* produces hydrogen sulfide by metabolizing sulfate, which activates the NF-κB inflammatory pathway and induces intestinal mucosal injury (60). In summary, gamma-linolenic acid and alpha-linolenic acid may negatively regulate *Desulfovibrio*, which damages intestinal mucosa through pro-inflammatory mechanisms, by inhibiting inflammatory signaling pathways.

This study has two notable limitations that should be considered when interpreting the results. Firstly, fecal samples were only collected at two time points (28 days and 35 days of age), which may not fully capture the dynamic succession of gut microbiota over the extended post-weaning period. Secondly, the sample size in the post-weaning diarrheic group (HD-D35, *n* = 17) was smaller than that in the post-weaning healthy group (HH-D35, *n* = 25). Although non-parametric statistical methods (Wilcoxon rank-sum test) were used to mitigate bias from unequal sample sizes, this imbalance have slightly affected the precision of statistical inferences (Supplementary Figure S5). Future studies with larger, balanced cohorts and longitudinal sampling designs are warranted to validate the findings.

## 5 Conclusion

This study systematically reveals the dynamic succession and structure of the gut microbiota and metabolites in diarrhea and healthy piglets pre-weaning and post-weaning. Weaning stress significantly altered microbial diversity (e.g., Shannon index changes) and community structure, with diarrhea further exacerbating dysbiosis. Metabolomics identified over a thousand differential metabolites involved in pathways such as amino acid and glycerophospholipid metabolism. Metabolites like flurandrenolide were positively associated with *Bacteroides* but negatively with *Holdemanella*. The study reveals weaning disrupts gut homeostasis and diarrhea amplifies metabolic disorder. The results of this study provide a reference for the prevention and treatment of diarrhea after weaning.

## Data Availability

The datasets generated in this study can be found in online repositories. The names of the repository/repositories and accession number(s) can be found at: https://doi.org/10.6084/m9.figshare.28784918.v1.

## Footnotes

^1^ http://www.kegg.jp

^2^ http://www.metaboanalyst.ca/
