# Supplementary material for: Comparison of fecal microbiota and metabolites in diarrheal piglets pre-and post-weaning
Source: Front Vet Sci. 2025 Jun 20;12:1613054. doi: 10.3389/fvets.2025.1613054 (PMC12226869; doi:10.3389/fvets.2025.1613054)
Supplement: Supplementary file 1 [file Supplementary_file_1.docx]

Supplementary Material

Comparison of fecal microbiota and metabolome in diarrheal piglets pre- and post-weaning

Tao Huang^1†^, Jiangpeng Dong^1†^, Wenyu Zhang^1^, Zhengyu Hu^1^, Xuhui Tan^1^, Hao Li^1^, Kailing Sun^1^, Ayong Zhao^1*^, and Min Huang^1*^

^1^Key Laboratory of Applied Technology on Green-Eco-Healthy Animal Husbandry of Zhejiang Province, College of Animal Science and Technology, College of Veterinary Medicine, Zhejiang A&F University, Hangzhou, China

*** Correspondence:** Ayong Zhao: [zay503@zafu.edu.cn](mailto:zay503@zafu.edu.cn); Min Huang: MinHuang0702@outlook.com

# Supplementary Figures and Tables

## Supplementary Figures


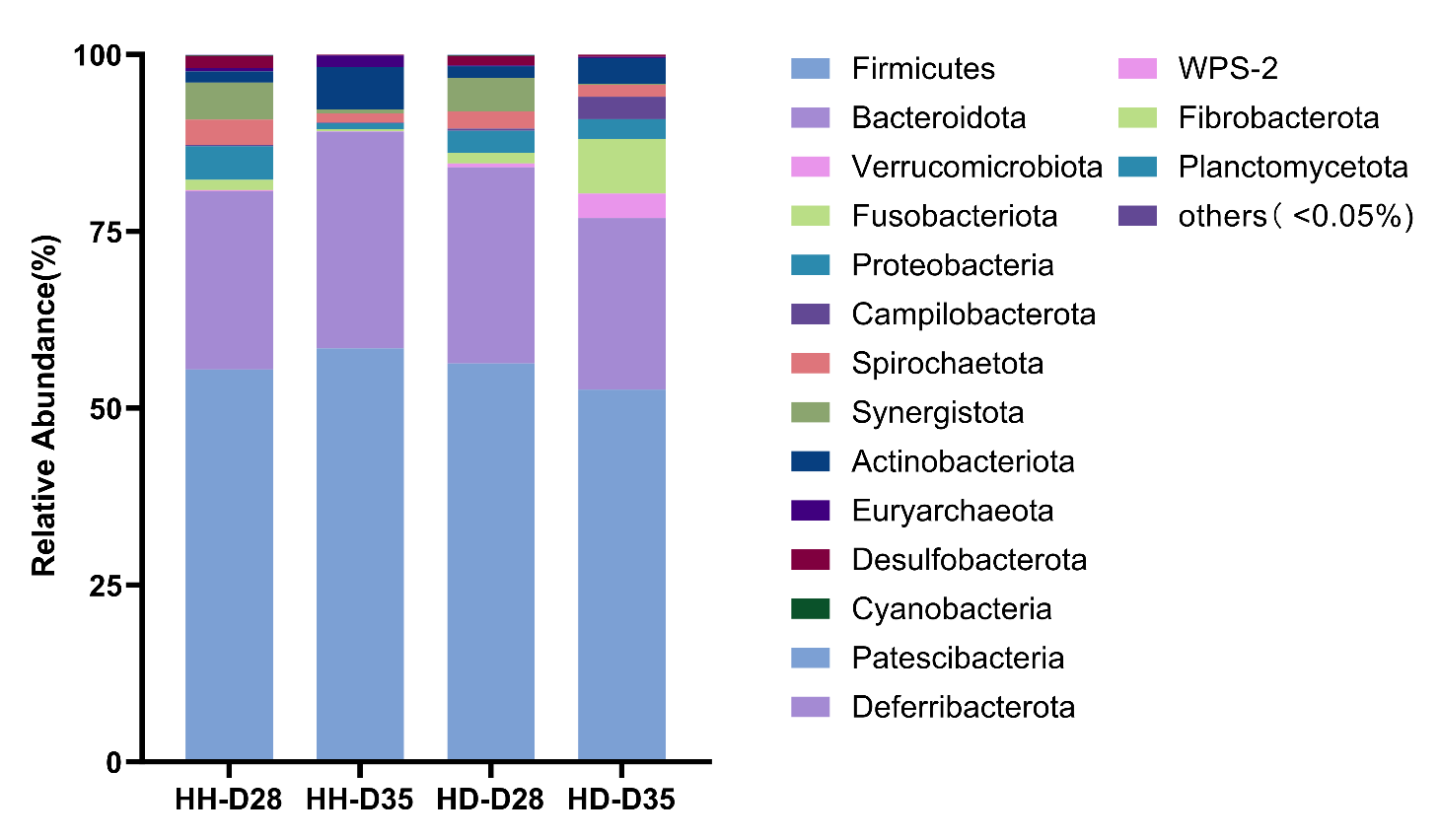


**Supplementary Figure 1.** Comparison of the relative abundance of phyla.

**
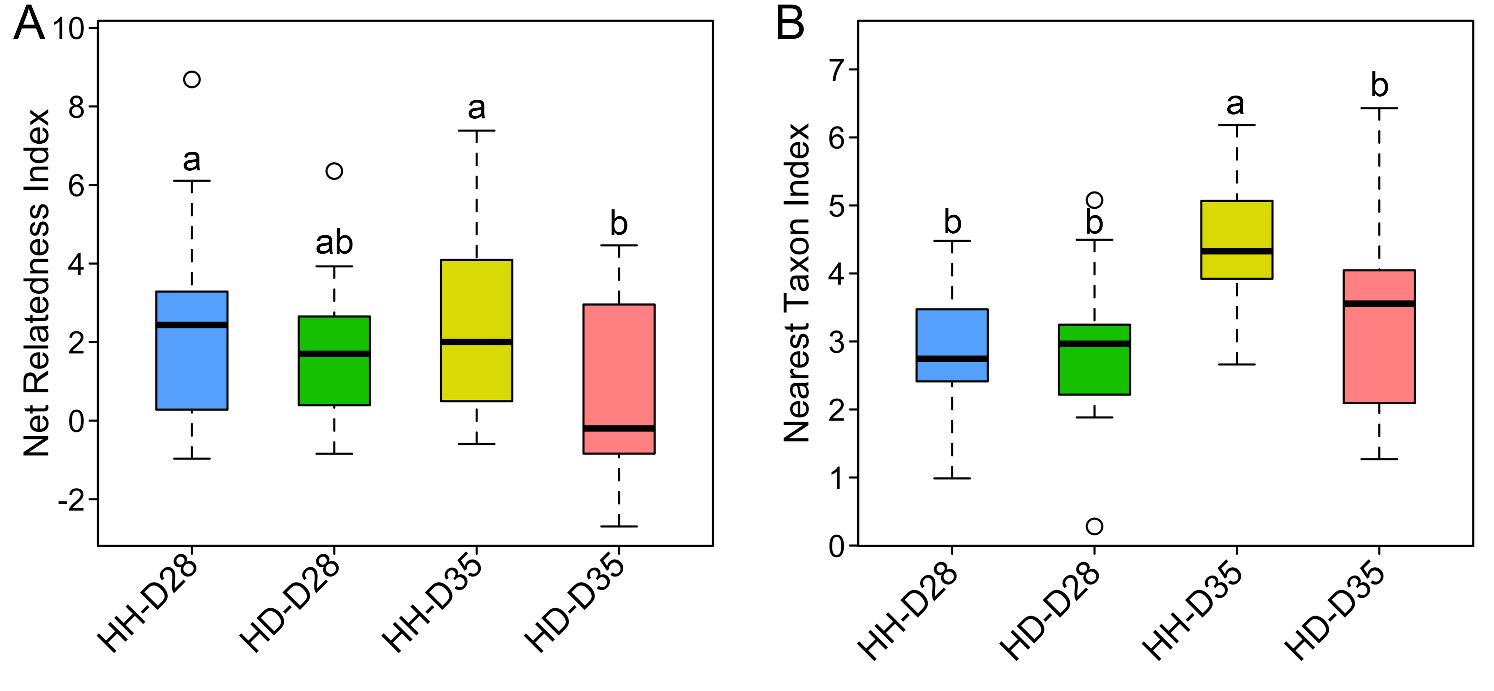
**

**Supplementary Figure 2.** Comparison of the net relatedness index and nearest taxon index.


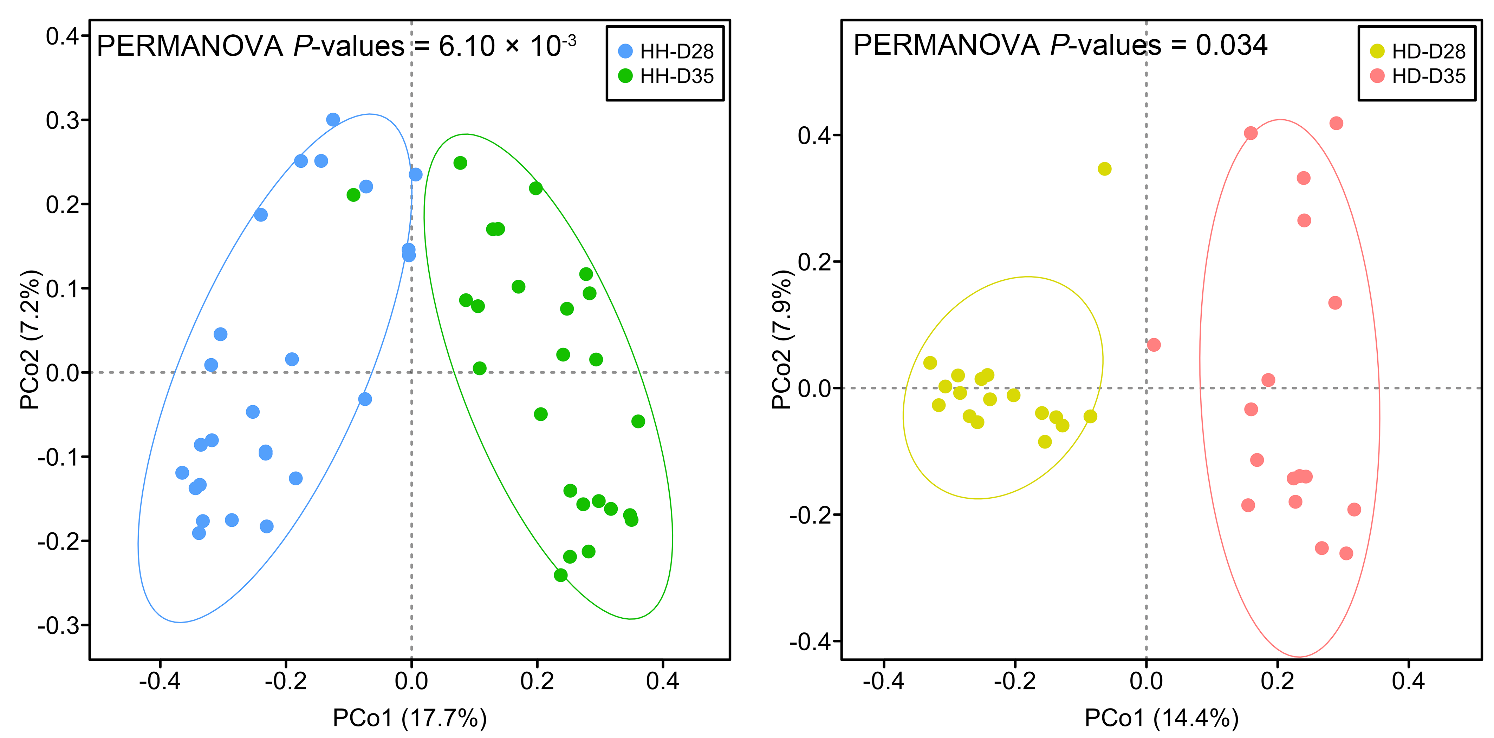


**Supplementary Figure 3.** Structure of fecal microbiota. (A) Principal coordinates analysis (PCoA) of fecal microbial communities between HH-D28 and HH-D35 groups. (B) Principal coordinates analysis (PCoA) of fecal microbial communities between HD-D28 and HD-D35 groups.


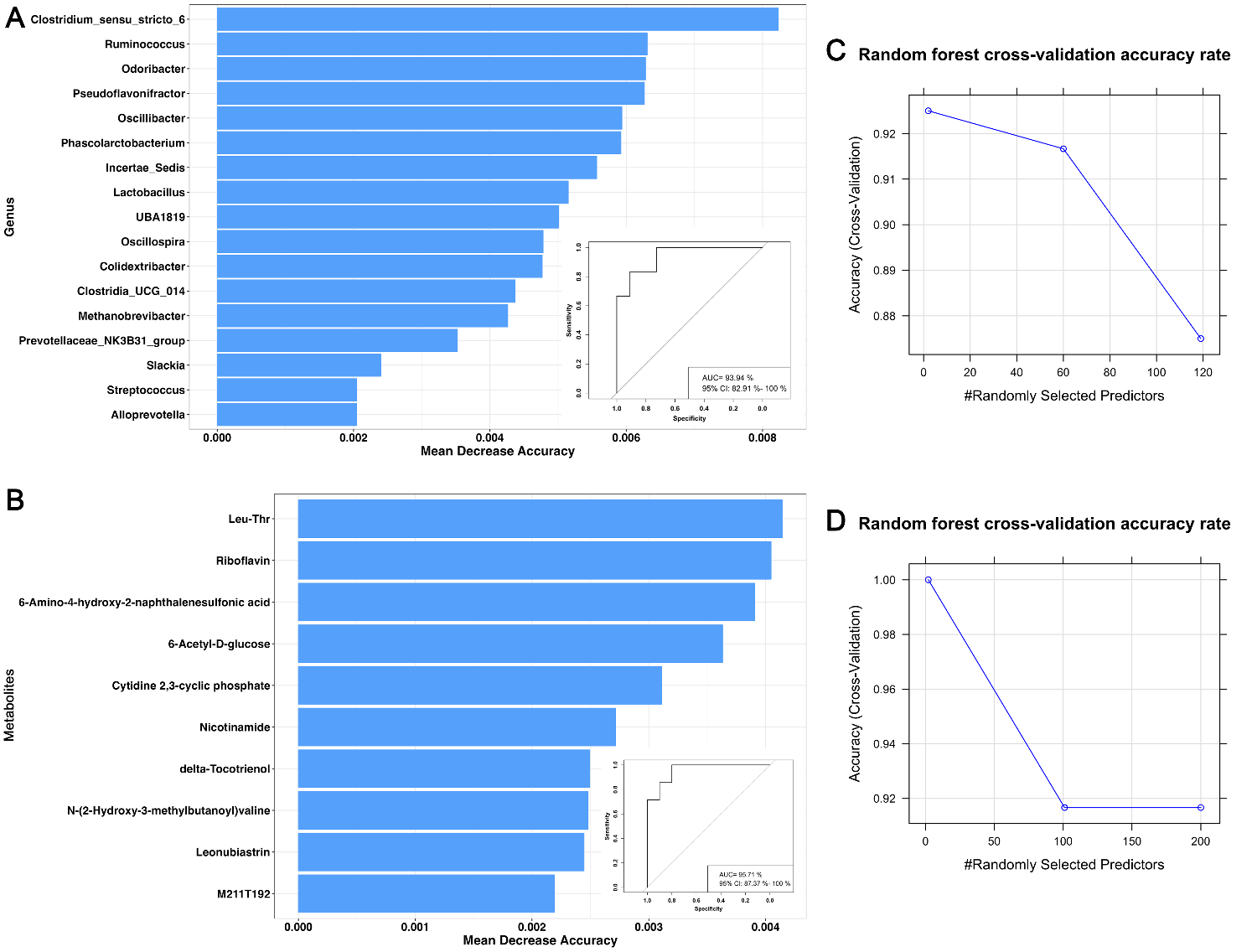


**Supplementary Figure 4.** Biomarker of bacterial genera and metabolites. (A) The top 17 bacterial genera biomarkers. (B) The top 10 metabolites biomarkers. (C) Random forest cross-validation accuracy rate of bacterial genera biomarkers. (D) Random forest cross-validation accuracy rate of metabolites biomarkers.


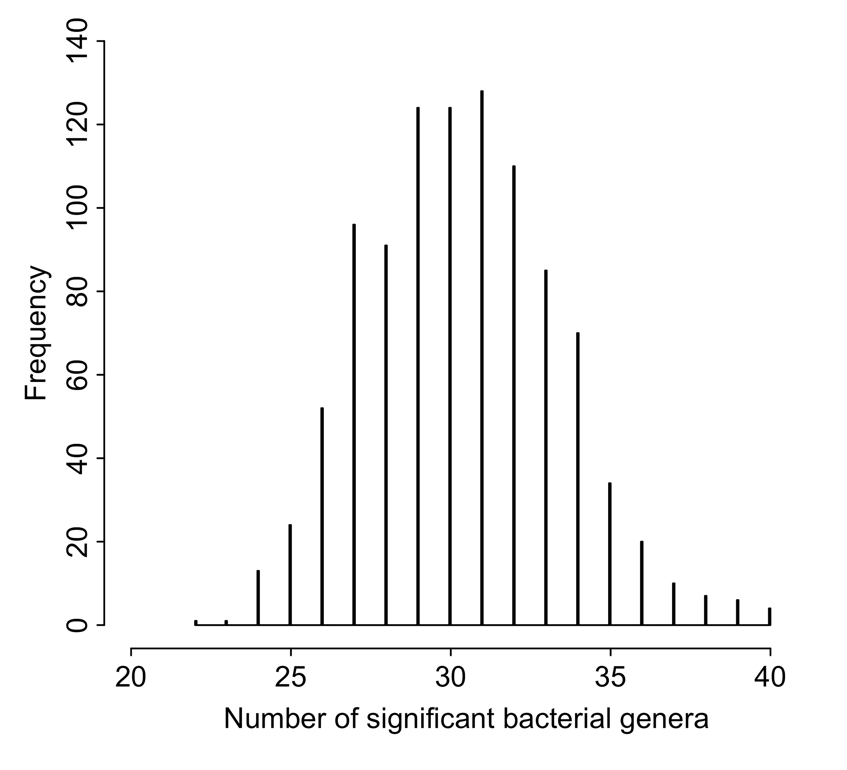


**Supplementary Figure 5.** Distribution of the number of significantly differentially genera in random sampling.

## Supplementary Tables

**Supplementary Table 1.** Summary of samples, sequencing data, and microbial structure.

| **Age** | **Sample number** | **Sequence number** | **ASVs passed quality control** | **Annotated phylum** | **Annotated genus** |
| --- | --- | --- | --- | --- | --- |
| D28 | 25 | 68399.76 ± 3893.05 | 2634 | 19 | 276 |
| D35 | 25 | 68874.76 ± 2732.15 | 2718 | 18 | 283 |
| D28 | 17 | 66087.36 ± 9699.33 | 2391 | 20 | 260 |
| D35 | 17 | 64034.47 ± 10760.07 | 2543 | 18 | 286 |

**Supplementary Table 2.** The composition of gut microbiota for each group at the genus level.

| **Genus** | **Relative abundance (%)** | | | | | ***p*-value** | ***p*-value (corrected)** |
| --- | --- | --- | --- | --- | --- | --- | --- |
|  | **All** | **HH-D28** | **HH-D35** | **HD-D28** | **HD-D35** |  |  |
| *Muribaculaceae* | 5.99±7.11 | 3.75±6.4 | 10.37±8.17 | 4.11±6.11 | 4.73±4.53 | 2.32E-03 | 2.32E-03 |
| *UCG-002* | 5.56±6.51 | 9.86±7.14 | 1.99±1.81 | 8.71±7.43 | 1.32±2.26 | 9.66E-08 | 9.66E-08 |
| *Bacteroides* | 5.4±7.94 | 9.86±10.04 | 0.75±1.02 | 9.82±8.14 | 1.25±2.04 | 9.44E-07 | 9.44E-07 |
| *Prevotella* | 4.16±7.57 | 0.98±1.43 | 7.22±6.77 | 1.82±2.62 | 6.68±13.27 | 0.01 | 0.01 |
| *Eubacterium_coprostanoligenes_group* | 4.04±3.56 | 6.17±4.61 | 3.2±2.21 | 3.67±2.73 | 2.48±2.85 | 2.16E-03 | 2.16E-03 |
| *Subdoligranulum* | 3.79±5.33 | 3.91±5.95 | 4.9±4.94 | 3.81±6.84 | 1.95±2.35 | 0.38 | 0.38 |
| *Lactobacillus* | 3.49±6.87 | 4.03±3.85 | 0.92±1.78 | 3.44±2.54 | 6.54±13.81 | 0.07 | 0.07 |
| *Christensenellaceae_R-7_group* | 3.44±4.5 | 5.65±5.66 | 2.08±3.23 | 3.82±3.7 | 1.81±3.79 | 0.01 | 0.01 |
| *Prevotellaceae_NK3B31_group* | 3.03±5.78 | 0.53±0.95 | 8.2±8.36 | 0.55±0.86 | 1.59±2.51 | 1.71E-07 | 1.71E-07 |
| *Rikenellaceae_RC9_gut_group* | 2.57±3.58 | 3.68±4.32 | 1.16±1.3 | 3.48±4.3 | 2.13±3.35 | 0.05 | 0.05 |
| *Lachnoclostridium* | 2.5±3.17 | 4.08±3.59 | 1.53±1.96 | 3.12±4.19 | 1.01±1.2 | 3.49E-03 | 3.49E-03 |
| *Fusobacterium* | 2.38±7.08 | 1.45±4.26 | 0.27±0.86 | 1.49±4.5 | 7.72±13.12 | 4.18E-03 | 4.18E-03 |
| *Treponema* | 2.25±4.68 | 3.56±6.03 | 1.31±1.71 | 2.2±5.99 | 1.75±3.82 | 0.37 | 0.37 |
| *Collinsella* | 2.23±2.87 | 1.06±1.68 | 3.9±3.8 | 1.11±1.99 | 2.61±2.19 | 8.14E-04 | 8.14E-04 |
| *Parabacteroides* | 2.2±2.31 | 3.42±2.84 | 1.02±1.33 | 2.66±1.7 | 1.69±2.26 | 1.12E-03 | 1.12E-03 |
| *Holdemanella* | 2.06±4.4 | 0.37±0.59 | 3.43±3.86 | 0.18±0.15 | 4.39±7.8 | 1.98E-03 | 1.98E-03 |
| *Escherichia-Shigella* | 2.04±4.75 | 3.84±7.12 | 0.44±1.04 | 2.24±3.17 | 1.55±4.48 | 0.08 | 0.08 |
| *Clostridium_sensu_stricto_1* | 1.87±2.83 | 1.6±2.01 | 2.04±2.84 | 1.89±3.15 | 2±3.66 | 0.95 | 0.95 |
| *Blautia* | 1.72±4.26 | 0.3±0.29 | 2.34±2.58 | 0.39±0.86 | 4.24±8.42 | 0.01 | 0.01 |
| *Pyramidobacter* | 1.64±3.53 | 3.15±4.2 | 0.02±0.05 | 3.41±4.94 | 0.01±0.02 | 2.38E-04 | 2.38E-04 |
| *UCG-005* | 1.63±2.33 | 1.12±1.71 | 2.22±2.63 | 2.05±2.93 | 1.07±1.81 | 0.23 | 0.23 |
| *Clostridia_UCG-014* | 1.33±2.45 | 1.56±2.67 | 1.17±1.83 | 1.73±3.68 | 0.81±1.17 | 0.68 | 0.68 |
| *Phascolarctobacterium* | 1.31±1.98 | 0.8±0.77 | 2±2.47 | 1.49±2.62 | 0.89±1.46 | 0.13 | 0.13 |
| *Alloprevotella* | 1.29±3.06 | 0.52±1.78 | 0.5±0.59 | 0.58±1.56 | 4.28±5.37 | 4.40E-05 | 4.40E-05 |
| *Ruminococcus* | 1.09±1.77 | 1.75±2.56 | 0.86±1.1 | 0.73±0.82 | 0.85±1.72 | 0.18 | 0.18 |
| *NK4A214_group* | 0.95±1.47 | 1.06±1.06 | 1.25±2.29 | 0.88±0.94 | 0.4±0.5 | 0.31 | 0.31 |
| *Family_XIII_AD3011_group* | 0.93±1.65 | 0.59±0.73 | 1.26±1.76 | 0.89±1.12 | 1.01±2.67 | 0.56 | 0.56 |
| *Cloacibacillus* | 0.8±1.94 | 1.52±2.11 | 0.55±2.61 | 0.8±1.05 | 0.11±0.37 | 0.11 | 0.11 |
| *Campylobacter* | 0.76±3.55 | 0.15±0.45 | 0.05±0.09 | 0.31±0.66 | 3.14±7.55 | 0.02 | 0.02 |
| *Akkermansia* | 0.76±6.43 | 0.1±0.35 | 0.07±0.18 | 0.00±0.01 | 3.49±14.29 | 0.28 | 0.28 |
| *Ruminococcus_torques_group* | 0.74±1.05 | 0.32±0.37 | 0.97±0.92 | 0.23±0.27 | 1.54±1.7 | 1.09E-04 | 1.09E-04 |
| *Catenisphaera* | 0.71±1.51 | 0.17±0.15 | 1.51±2.4 | 0.53±0.93 | 0.51±0.86 | 0.01 | 0.01 |
| *Alistipes* | 0.7±2.4 | 0.88±0.82 | 0.09±0.24 | 1.99±5.1 | 0.07±0.09 | 0.05 | 0.05 |
| *Methanobrevibacter* | 0.7±1.92 | 0.49±1.24 | 1.66±3.1 | 0.13±0.19 | 0.15±0.31 | 0.02 | 0.02 |
| *Lachnospiraceae_NK4A136_group* | 0.65±1.9 | 0.18±0.24 | 1.42±3.23 | 0.1±0.12 | 0.75±1.21 | 0.06 | 0.06 |
| *Megasphaera* | 0.64±2.73 | 0.38±0.88 | 0.54±1.41 | 0.32±0.69 | 1.49±5.74 | 0.55 | 0.55 |
| *RF39* | 0.62±1.71 | 0.45±1.26 | 1.19±2.72 | 0.3±0.56 | 0.35±0.78 | 0.26 | 0.26 |
| *Roseburia* | 0.61±1.41 | 0.32±1.11 | 0.92±1.63 | 0.21±0.49 | 1.01±1.89 | 0.17 | 0.17 |
| *Faecalibacterium* | 0.61±1.55 | 0.04±0.07 | 1.18±2.18 | 0.11±0.13 | 1.1±1.94 | 0.01 | 0.01 |
| *Desulfovibrio* | 0.59±0.72 | 1.1±0.77 | 0.08±0.17 | 0.95±0.64 | 0.23±0.48 | 3.41E-09 | 3.41E-09 |
| *Romboutsia* | 0.57±1.55 | 0.94±1.32 | 0.05±0.14 | 1.36±2.86 | 0.02±0.03 | 0.01 | 0.01 |
| *Olsenella* | 0.55±1.1 | 0.12±0.49 | 1.36±1.56 | 0.2±0.69 | 0.31±0.59 | 6.86E-05 | 6.86E-05 |
| *Ruminococcus_gauvreauii_group* | 0.53±1.22 | 0.32±0.45 | 0.9±2.05 | 0.2±0.24 | 0.61±0.81 | 0.22 | 0.22 |
| *Agathobacter* | 0.48±1.55 | 0.02±0.04 | 0.68±1.56 | 0.03±0.08 | 1.29±2.75 | 0.03 | 0.03 |
| *Coprococcus* | 0.45±0.92 | 0.06±0.07 | 0.88±1.43 | 0.23±0.33 | 0.6±0.77 | 0.01 | 0.01 |
| *Colidextribacter* | 0.45±0.65 | 0.42±0.46 | 0.75±1.01 | 0.3±0.21 | 0.18±0.22 | 0.03 | 0.03 |
| *Terrisporobacter* | 0.41±0.76 | 0.55±1.13 | 0.47±0.53 | 0.38±0.68 | 0.15±0.29 | 0.39 | 0.39 |
| *Solobacterium* | 0.4±1.41 | 0.01±0.02 | 1.17±2.43 | 0.02±0.04 | 0.22±0.3 | 0.01 | 0.01 |
| *UCG-010* | 0.4±0.46 | 0.46±0.44 | 0.36±0.41 | 0.52±0.65 | 0.22±0.31 | 0.24 | 0.24 |
| *Succinivibrio* | 0.39±1.52 | 0.53±1.45 | 0.24±0.63 | 0.73±2.8 | 0.05±0.09 | 0.54 | 0.54 |
| *Tyzzerella* | 0.37±0.73 | 0.14±0.21 | 0.54±1.05 | 0.39±0.74 | 0.44±0.61 | 0.28 | 0.28 |
| *Streptococcus* | 0.32±0.92 | 0.21±0.24 | 0.47±1.5 | 0.3±0.85 | 0.29±0.42 | 0.78 | 0.78 |
| *p-2534-18B5_gut_group* | 0.31±0.98 | 0.22±0.48 | 0.00±0.00 | 1.14±1.9 | 0.04±0.07 | 4.89E-04 | 4.89E-04 |
| *Succiniclasticum* | 0.3±2.21 | 0.05±0.08 | 0.01±0.04 | 1.36±4.88 | 0.01±0.02 | 0.17 | 0.17 |
| *Butyricimonas* | 0.28±0.43 | 0.51±0.5 | 0.05±0.17 | 0.56±0.49 | 0.02±0.03 | 6.50E-07 | 6.50E-07 |
| *UCG-008* | 0.22±0.53 | 0.03±0.07 | 0.44±0.71 | 0.01±0.01 | 0.41±0.7 | 4.62E-03 | 4.62E-03 |
| *Bilophila* | 0.22±0.39 | 0.48±0.54 | 0.02±0.03 | 0.3±0.3 | 0.06±0.17 | 1.51E-05 | 1.51E-05 |
| *Peptostreptococcus* | 0.22±1.73 | 0.00±0.00 | 0.03±0.08 | 0.00±0.01 | 1.04±3.81 | 0.19 | 0.19 |
| *Negativibacillus* | 0.21±0.36 | 0.08±0.18 | 0.32±0.32 | 0.31±0.63 | 0.12±0.11 | 0.04 | 0.04 |
| *Eubacterium_eligens_group* | 0.2±0.57 | 0.04±0.14 | 0.35±0.49 | 0.01±0.02 | 0.43±1.06 | 0.04 | 0.04 |
| *Catenibacterium* | 0.2±0.99 | 0.05±0.15 | 0.17±0.21 | 0.02±0.04 | 0.64±2.16 | 0.21 | 0.21 |
| *Prevotellaceae_UCG-003* | 0.2±0.45 | 0.07±0.16 | 0.29±0.6 | 0.02±0.03 | 0.42±0.61 | 0.02 | 0.02 |
| *Fournierella* | 0.19±0.46 | 0.09±0.32 | 0.2±0.45 | 0.35±0.7 | 0.17±0.33 | 0.36 | 0.36 |
| *Mogibacterium* | 0.19±0.65 | 0.35±1.17 | 0.09±0.11 | 0.23±0.22 | 0.06±0.1 | 0.41 | 0.41 |
| *Dorea* | 0.18±0.61 | 0.01±0.01 | 0.13±0.16 | 0.00±0.01 | 0.69±1.24 | 8.04E-04 | 8.04E-04 |
| *Incertae_Sedis* | 0.18±0.57 | 0.04±0.07 | 0.49±0.96 | 0.01±0.01 | 0.1±0.24 | 0.01 | 0.01 |
| *Eubacterium_siraeum_group* | 0.18±0.45 | 0.03±0.08 | 0.46±0.74 | 0.01±0.02 | 0.13±0.21 | 1.12E-03 | 1.12E-03 |
| *hoa5-07d05_gut_group* | 0.17±0.74 | 0.07±0.19 | 0.14±0.34 | 0.02±0.03 | 0.54±1.55 | 0.14 | 0.14 |
| *Butyricicoccus* | 0.16±0.24 | 0.08±0.12 | 0.19±0.23 | 0.06±0.08 | 0.31±0.38 | 4.04E-03 | 4.04E-03 |
| *T34* | 0.15±0.51 | 0.01±0.01 | 0.09±0.26 | 0.00±0.01 | 0.61±0.99 | 2.65E-04 | 2.65E-04 |
| *UBA1819* | 0.14±0.2 | 0.13±0.12 | 0.2±0.21 | 0.09±0.11 | 0.11±0.3 | 0.28 | 0.28 |
| *CHKCI001* | 0.14±0.77 | 0.03±0.09 | 0.00±0.01 | 0.62±1.67 | 0.00±0.00 | 0.04 | 0.04 |
| *Erysipelotrichaceae_UCG-009* | 0.13±0.53 | 0.24±0.87 | 0.11±0.28 | 0.02±0.06 | 0.13±0.43 | 0.62 | 0.62 |
| *Oscillibacter* | 0.13±0.19 | 0.12±0.17 | 0.24±0.27 | 0.09±0.11 | 0.04±0.05 | 2.67E-03 | 2.67E-03 |
| *Prevotellaceae_UCG-004* | 0.12±0.21 | 0.14±0.22 | 0.1±0.21 | 0.21±0.26 | 0.05±0.09 | 0.15 | 0.15 |
| *Monoglobus* | 0.12±0.19 | 0.1±0.14 | 0.14±0.2 | 0.18±0.28 | 0.08±0.12 | 0.43 | 0.43 |
| *Clostridium_sensu_stricto_6* | 0.12±0.33 | 0.02±0.04 | 0.36±0.53 | 0.00±0.01 | 0.04±0.03 | 9.40E-05 | 9.40E-05 |
| *Synergistes* | 0.12±0.33 | 0.2±0.34 | 0.00±0.01 | 0.3±0.57 | 0.00±0.00 | 0.01 | 0.01 |
| *Candidatus_Soleaferrea* | 0.12±0.27 | 0.05±0.09 | 0.18±0.37 | 0.14±0.34 | 0.11±0.18 | 0.44 | 0.44 |
| *Flavonifractor* | 0.11±0.62 | 0.02±0.03 | 0.34±1.11 | 0.03±0.07 | 0.02±0.02 | 0.21 | 0.21 |
| *Odoribacter* | 0.11±0.41 | 0.15±0.46 | 0.01±0.02 | 0.15±0.27 | 0.18±0.67 | 0.48 | 0.48 |
| *Fusicatenibacter* | 0.11±0.41 | 0.18±0.68 | 0.07±0.08 | 0.01±0.01 | 0.17±0.37 | 0.50 | 0.50 |
| *Peptococcus* | 0.11±0.24 | 0.07±0.18 | 0.08±0.23 | 0.27±0.36 | 0.04±0.08 | 0.01 | 0.01 |
| *Intestinimonas* | 0.11±0.15 | 0.11±0.12 | 0.06±0.07 | 0.19±0.24 | 0.07±0.1 | 0.02 | 0.02 |
| *Oribacterium* | 0.1±0.36 | 0.09±0.34 | 0.18±0.57 | 0.02±0.02 | 0.09±0.1 | 0.56 | 0.56 |
| *Lachnospiraceae_FCS020_group* | 0.1±0.21 | 0.04±0.04 | 0.13±0.16 | 0.16±0.4 | 0.08±0.11 | 0.22 | 0.22 |
| *Oscillospira* | 0.1±0.19 | 0.02±0.03 | 0.18±0.19 | 0.11±0.28 | 0.06±0.15 | 0.01 | 0.01 |
| *Actinobacillus* | 0.09±0.53 | 0.01±0.01 | 0.02±0.06 | 0.01±0.01 | 0.43±1.14 | 0.04 | 0.04 |
| *Chlamydia* | 0.09±0.66 | 0.00±0.01 | 0.00±0.01 | 0.45±1.45 | 0.00±0.00 | 0.11 | 0.11 |
| *dgA-11_gut_group* | 0.09±0.33 | 0.06±0.09 | 0.01±0.03 | 0.27±0.7 | 0.05±0.07 | 0.07 | 0.07 |
| *Sharpea* | 0.09±0.49 | 0.23±0.89 | 0.04±0.11 | 0.03±0.05 | 0.02±0.04 | 0.41 | 0.41 |
| *Sellimonas* | 0.09±0.21 | 0.11±0.17 | 0.05±0.12 | 0.19±0.37 | 0.01±0.01 | 0.04 | 0.04 |
| *Anaerostipes* | 0.09±0.42 | 0.00±0.01 | 0.24±0.74 | 0.00±0.00 | 0.06±0.08 | 0.15 | 0.15 |
| *Ruminococcus_gnavus_group* | 0.08±0.53 | 0.01±0.02 | 0.05±0.1 | 0.01±0.03 | 0.32±1.17 | 0.24 | 0.24 |
| *Anaerovibrio* | 0.08±0.3 | 0.03±0.1 | 0.11±0.42 | 0.03±0.08 | 0.16±0.42 | 0.46 | 0.46 |
| *Eubacterium_nodatum_group* | 0.08±0.17 | 0.11±0.13 | 0.07±0.26 | 0.05±0.09 | 0.09±0.13 | 0.71 | 0.71 |
| *Intestinibacter* | 0.08±0.2 | 0.04±0.06 | 0.21±0.33 | 0.03±0.05 | 0.01±0.02 | 2.13E-03 | 2.13E-03 |
| *Acidaminococcus* | 0.08±0.19 | 0.08±0.16 | 0.08±0.13 | 0.14±0.34 | 0.02±0.03 | 0.29 | 0.29 |
| *Eubacterium_hallii_group* | 0.08±0.15 | 0.03±0.04 | 0.16±0.25 | 0.03±0.05 | 0.06±0.07 | 0.01 | 0.01 |
| *Eubacterium_ruminantium_group* | 0.07±0.44 | 0.01±0.03 | 0.21±0.79 | 0.00±0.00 | 0.04±0.09 | 0.31 | 0.31 |
| *Eubacterium_fissicatena_group* | 0.07±0.1 | 0.09±0.09 | 0.04±0.05 | 0.14±0.16 | 0.03±0.05 | 9.31E-04 | 9.31E-04 |
| *Lachnospiraceae_UCG-002* | 0.07±0.19 | 0.11±0.25 | 0.01±0.02 | 0.11±0.2 | 0.07±0.21 | 0.23 | 0.23 |
| *Sutterella* | 0.07±0.43 | 0.2±0.79 | 0.01±0.02 | 0.03±0.03 | 0.01±0.01 | 0.35 | 0.35 |
| *Syntrophococcus* | 0.07±0.28 | 0.03±0.1 | 0.18±0.49 | 0.01±0.01 | 0.05±0.08 | 0.17 | 0.17 |
| *Clostridia_vadinBB60_group* | 0.07±0.12 | 0.08±0.1 | 0.02±0.03 | 0.13±0.16 | 0.07±0.15 | 0.03 | 0.03 |
| *Dialister* | 0.07±0.58 | 0.01±0.01 | 0.22±1.06 | 0.01±0.02 | 0.01±0.01 | 0.52 | 0.52 |
| *Pseudoflavonifractor* | 0.07±0.14 | 0.07±0.11 | 0.06±0.08 | 0.09±0.17 | 0.05±0.19 | 0.82 | 0.82 |
| *Anaerorhabdus_furcosa_group* | 0.07±0.11 | 0.07±0.11 | 0.09±0.14 | 0.07±0.1 | 0.02±0.04 | 0.33 | 0.33 |
| *Sediminispirochaeta* | 0.06±0.43 | 0.04±0.13 | 0.00±0.00 | 0.24±0.95 | 0.00±0.00 | 0.30 | 0.30 |
| *Lachnospiraceae_AC2044_group* | 0.06±0.21 | 0.02±0.05 | 0.09±0.22 | 0.00±0.01 | 0.12±0.38 | 0.22 | 0.22 |
| *UCG-009* | 0.06±0.11 | 0.05±0.1 | 0.09±0.15 | 0.05±0.09 | 0.04±0.04 | 0.47 | 0.47 |
| *Bradymonadales* | 0.05±0.24 | 0.12±0.44 | 0.01±0.03 | 0.06±0.1 | 0.01±0.01 | 0.35 | 0.35 |
| *Lachnospiraceae* | 0.05±0.18 | 0.01±0.04 | 0.02±0.03 | 0.00±0.00 | 0.21±0.37 | 6.08E-04 | 6.08E-04 |
| *Slackia* | 0.05±0.09 | 0.01±0.01 | 0.1±0.1 | 0.01±0.01 | 0.09±0.12 | 7.12E-06 | 7.12E-06 |
| *Lachnospiraceae_XPB1014_group* | 0.05±0.22 | 0.1±0.39 | 0.05±0.12 | 0.01±0.02 | 0.03±0.08 | 0.62 | 0.62 |
| *Gastranaerophilales* | 0.05±0.18 | 0.09±0.23 | 0.00±0.00 | 0.11±0.26 | 0.01±0.01 | 0.09 | 0.09 |
| *Enterorhabdus* | 0.05±0.08 | 0.02±0.05 | 0.1±0.11 | 0.03±0.06 | 0.04±0.06 | 6.21E-04 | 6.21E-04 |
| *Enterococcus* | 0.05±0.15 | 0.06±0.11 | 0.01±0.01 | 0.12±0.29 | 0.03±0.08 | 0.09 | 0.09 |
| *Eisenbergiella* | 0.05±0.18 | 0.07±0.13 | 0.07±0.31 | 0.04±0.04 | 0.01±0.01 | 0.68 | 0.68 |
| *others(<0.05%)* | 6.88±5.11 | 5.54±3.49 | 7.19±4.46 | 9.54±8.1 | 5.67±3.12 | 0.57 | 0.57 |

**Supplementary Table 3.** The different enrichments of KEGG pathways between groups.

| **KEGG Pathways** | **HH-D28** | | **HH-D35** | | ***P*-values** | ***P*-values (corrected)** | **Difference between means** |
| --- | --- | --- | --- | --- | --- | --- | --- |
|  | **Mean (%)** | **SD (%)** | **Mean (%)** | **SD (%)** |  |  |  |
| Epithelial cell signaling in Helicobacter pylori infection | 0.14 | 0.02 | 0.17 | 0.01 | 4.15E-09 | 6.93E-07 | -0.03 |
| Carbon fixation pathways in prokaryotes | 1.19 | 0.06 | 1.08 | 0.06 | 2.62E-08 | 2.19E-06 | 0.11 |
| Alanine, aspartate and glutamate metabolism | 1.72 | 0.06 | 1.82 | 0.05 | 6.39E-08 | 2.67E-06 | -0.10 |
| Carbon fixation in photosynthetic organisms | 1.61 | 0.06 | 1.75 | 0.08 | 5.35E-08 | 2.98E-06 | -0.14 |
| Nicotinate and nicotinamide metabolism | 1.10 | 0.05 | 1.20 | 0.06 | 1.58E-07 | 5.27E-06 | -0.10 |
| Nucleotide excision repair | 0.80 | 0.05 | 0.87 | 0.03 | 1.91E-07 | 5.32E-06 | -0.07 |
| D-Glutamine and D-glutamate metabolism | 2.23 | 0.15 | 2.44 | 0.09 | 6.26E-07 | 1.49E-05 | -0.21 |
| Terpenoid backbone biosynthesis | 1.38 | 0.08 | 1.49 | 0.05 | 9.00E-07 | 1.50E-05 | -0.11 |
| Starch and sucrose metabolism | 0.96 | 0.06 | 1.06 | 0.06 | 7.54E-07 | 1.57E-05 | -0.10 |
| Citrate cycle (TCA cycle) | 1.01 | 0.08 | 0.87 | 0.09 | 8.84E-07 | 1.64E-05 | 0.14 |
| Aminobenzoate degradation | 0.13 | 0.03 | 0.09 | 0.02 | 1.28E-06 | 1.94E-05 | 0.04 |
| Two-component system | 0.42 | 0.04 | 0.35 | 0.05 | 1.65E-06 | 2.30E-05 | 0.07 |
| Purine metabolism | 0.90 | 0.02 | 0.94 | 0.03 | 3.36E-06 | 3.74E-05 | -0.04 |
| Base excision repair | 0.92 | 0.05 | 0.99 | 0.03 | 3.04E-06 | 3.91E-05 | -0.07 |
| Naphthalene degradation | 0.15 | 0.11 | 0.02 | 0.05 | 3.29E-06 | 3.93E-05 | 0.14 |
| Valine, leucine and isoleucine degradation | 0.43 | 0.06 | 0.35 | 0.04 | 7.13E-06 | 7.44E-05 | 0.08 |
| NOD-like receptor signaling pathway | 0.08 | 8.51E-03 | 0.09 | 4.71E-03 | 1.20E-05 | 1.18E-04 | -0.01 |
| Toxoplasmosis | 2.64E-03 | 2.01E-03 | 4.21E-04 | 6.43E-04 | 1.66E-05 | 1.54E-04 | 2.22E-03 |
| Homologous recombination | 1.56 | 0.07 | 1.64 | 0.06 | 1.98E-05 | 1.74E-04 | -0.09 |
| Aminoacyl-tRNA biosynthesis | 1.76 | 0.11 | 1.89 | 0.07 | 2.26E-05 | 1.79E-04 | -0.12 |
| Amino sugar and nucleotide sugar metabolism | 1.08 | 0.07 | 1.18 | 0.08 | 2.21E-05 | 1.85E-04 | -0.10 |
| One carbon pool by folate | 1.77 | 0.07 | 1.91 | 0.12 | 2.71E-05 | 2.06E-04 | -0.14 |
| Selenocompound metabolism | 1.08 | 0.04 | 1.02 | 0.04 | 3.13E-05 | 2.09E-04 | 0.05 |
| Pyrimidine metabolism | 1.22 | 0.04 | 1.28 | 0.05 | 3.10E-05 | 2.16E-04 | -0.06 |
| Butanoate metabolism | 0.75 | 0.07 | 0.67 | 0.05 | 3.00E-05 | 2.18E-04 | 0.08 |
| Biotin metabolism | 1.28 | 0.20 | 1.05 | 0.15 | 3.89E-05 | 2.50E-04 | 0.23 |
| Ribosome | 1.68 | 0.10 | 1.79 | 0.05 | 4.63E-05 | 2.86E-04 | -0.11 |
| Phenylalanine, tyrosine and tryptophan biosynthesis | 1.17 | 0.08 | 1.26 | 0.05 | 5.23E-05 | 3.12E-04 | -0.09 |
| Fructose and mannose metabolism | 0.98 | 0.12 | 1.13 | 0.11 | 5.95E-05 | 3.43E-04 | -0.14 |
| DNA replication | 1.26 | 0.05 | 1.31 | 0.04 | 8.60E-05 | 4.79E-04 | -0.06 |
| Biosynthesis of vancomycin group antibiotics | 1.91 | 0.23 | 2.17 | 0.18 | 8.99E-05 | 4.84E-04 | -0.26 |
| Cell cycle - Caulobacter | 1.48 | 0.07 | 1.57 | 0.07 | 1.07E-04 | 5.59E-04 | -0.08 |
| Steroid hormone biosynthesis | 0.03 | 0.02 | 7.14E-03 | 5.02E-03 | 1.50E-04 | 7.58E-04 | 0.02 |
| Drug metabolism - other enzymes | 1.13 | 0.57 | 0.41 | 0.65 | 1.72E-04 | 8.47E-04 | 0.72 |
| Phenylalanine metabolism | 0.31 | 0.04 | 0.27 | 0.02 | 1.94E-04 | 9.26E-04 | 0.04 |
| Pantothenate and CoA biosynthesis | 1.79 | 0.08 | 1.86 | 0.04 | 2.01E-04 | 9.33E-04 | -0.07 |
| Mismatch repair | 1.74 | 0.08 | 1.82 | 0.06 | 2.31E-04 | 1.04E-03 | -0.08 |
| Flagellar assembly | 1.15 | 0.27 | 0.83 | 0.28 | 3.00E-04 | 1.32E-03 | 0.31 |
| Peptidoglycan biosynthesis | 1.98 | 0.17 | 2.13 | 0.05 | 3.15E-04 | 1.35E-03 | -0.15 |
| Neomycin, kanamycin and gentamicin biosynthesis | 0.01 | 0.06 | 0.14 | 0.14 | 4.62E-04 | 1.93E-03 | -0.12 |
| Cysteine and methionine metabolism | 1.37 | 0.11 | 1.46 | 0.04 | 5.21E-04 | 2.07E-03 | -0.09 |
| Histidine metabolism | 1.31 | 0.11 | 1.41 | 0.08 | 5.12E-04 | 2.08E-03 | -0.10 |
| Phosphotransferase system (PTS) | 0.37 | 0.18 | 0.57 | 0.20 | 6.03E-04 | 2.29E-03 | -0.20 |
| Synthesis and degradation of ketone bodies | 0.63 | 0.15 | 0.49 | 0.11 | 5.99E-04 | 2.32E-03 | 0.14 |
| Tetracycline biosynthesis | 0.30 | 0.35 | 0.02 | 0.11 | 8.25E-04 | 3.06E-03 | 0.28 |
| Glutathione metabolism | 0.42 | 0.08 | 0.35 | 0.04 | 1.01E-03 | 3.67E-03 | 0.07 |
| Chloroalkane and chloroalkene degradation | 0.23 | 0.21 | 0.05 | 0.14 | 1.11E-03 | 3.93E-03 | 0.18 |
| Galactose metabolism | 0.96 | 0.12 | 1.06 | 0.08 | 1.16E-03 | 4.03E-03 | -0.10 |
| Toluene degradation | 0.19 | 0.09 | 0.09 | 0.11 | 1.44E-03 | 4.91E-03 | 0.10 |
| Polyketide sugar unit biosynthesis | 0.19 | 0.22 | 0.02 | 0.11 | 1.86E-03 | 6.22E-03 | 0.17 |
| Tyrosine metabolism | 0.27 | 0.03 | 0.24 | 0.02 | 2.23E-03 | 7.31E-03 | 0.03 |
| Glycerolipid metabolism | 0.49 | 0.05 | 0.53 | 0.04 | 2.39E-03 | 7.69E-03 | -0.04 |
| Fatty acid degradation | 0.37 | 0.05 | 0.33 | 0.02 | 3.13E-03 | 9.86E-03 | 0.04 |
| Linoleic acid metabolism | 0.34 | 0.06 | 0.25 | 0.12 | 3.49E-03 | 0.011 | 0.09 |
| Carotenoid biosynthesis | 6.74E-03 | 7.13E-03 | 1.92E-03 | 1.86E-03 | 3.46E-03 | 0.011 | 4.82E-03 |
| Biosynthesis of ansamycins | 5.18 | 0.41 | 5.67 | 0.67 | 3.75E-03 | 0.011 | -0.49 |
| Hypertrophic cardiomyopathy (HCM) | 6.61E-05 | 8.94E-05 | 7.78E-06 | 1.45E-05 | 4.10E-03 | 0.012 | 5.83E-05 |
| Photosynthesis | 0.07 | 0.16 | 0.27 | 0.29 | 4.18E-03 | 0.012 | -0.20 |
| Dioxin degradation | 0.18 | 0.05 | 0.13 | 0.07 | 5.69E-03 | 0.016 | 0.05 |
| Zeatin biosynthesis | 0.67 | 0.06 | 0.73 | 0.09 | 5.89E-03 | 0.016 | -0.06 |
| Nitrotoluene degradation | 0.32 | 0.10 | 0.25 | 0.06 | 6.33E-03 | 0.017 | 0.07 |
| Propanoate metabolism | 0.75 | 0.04 | 0.70 | 0.06 | 6.28E-03 | 0.017 | 0.04 |
| beta-Lactam resistance (ko01501) | 0.18 | 0.06 | 0.26 | 0.12 | 7.05E-03 | 0.018 | -0.08 |
| Bacterial chemotaxis | 1.59 | 0.38 | 1.29 | 0.36 | 6.96E-03 | 0.018 | 0.31 |
| Systemic lupus erythematosus | 1.01E-05 | 1.68E-05 | 1.52E-07 | 7.45E-07 | 7.79E-03 | 0.020 | 9.98E-06 |
| Biofilm formation - Vibrio cholerae | 0.21 | 0.02 | 0.18 | 0.04 | 8.46E-03 | 0.021 | 0.03 |
| Lipoic acid metabolism | 0.84 | 0.15 | 0.68 | 0.24 | 0.011 | 0.027 | 0.15 |
| Pentose and glucuronate interconversions | 0.70 | 0.12 | 0.64 | 0.04 | 0.011 | 0.027 | 0.07 |
| RNA polymerase | 1.21 | 0.15 | 1.31 | 0.08 | 0.011 | 0.028 | -0.09 |
| Glycine, serine and threonine metabolism | 1.17 | 0.05 | 1.14 | 0.03 | 0.012 | 0.030 | 0.03 |
| N-Glycan biosynthesis | 0.04 | 0.01 | 0.05 | 0.02 | 0.013 | 0.030 | -0.01 |
| Glycolysis / Gluconeogenesis | 1.15 | 0.05 | 1.18 | 0.05 | 0.013 | 0.030 | -0.04 |
| Arginine and proline metabolism | 0.76 | 0.03 | 0.74 | 0.02 | 0.013 | 0.030 | 0.02 |
| D-Arginine and D-ornithine metabolism | 0.12 | 0.09 | 0.21 | 0.15 | 0.013 | 0.030 | -0.09 |
| Tryptophan metabolism | 0.20 | 0.02 | 0.18 | 0.02 | 0.013 | 0.030 | 0.02 |
| Fatty acid biosynthesis | 1.81 | 0.14 | 1.72 | 0.10 | 0.014 | 0.032 | 0.09 |
| Other types of O-glycan biosynthesis | 6.14E-05 | 8.97E-05 | 1.34E-05 | 2.15E-05 | 0.017 | 0.037 | 4.80E-05 |
| Lysine biosynthesis | 1.73 | 0.09 | 1.78 | 0.06 | 0.019 | 0.041 | -0.05 |
| Pentose phosphate pathway | 1.74 | 0.12 | 1.81 | 0.07 | 0.021 | 0.044 | -0.07 |
| Staphylococcus aureus infection | 0.03 | 0.01 | 0.02 | 0.01 | 0.022 | 0.045 | 9.06E-03 |
| Bisphenol degradation | 0.07 | 0.14 | 0 | 0 | 0.023 | 0.048 | 0.07 |

| **KEGG Pathways** | **HH-D35** | | **HD-D35** | | ***P*-values** | ***P*-values (corrected)** | **Difference between means** |
| --- | --- | --- | --- | --- | --- | --- | --- |
|  | **Mean (%)** | **SD (%)** | **Mean (%)** | **SD (%)** |  |  |  |
| Protein processing in endoplasmic reticulum | 0.06 | 8.24E-03 | 0.05 | 8.74E-03 | 1.07E-04 | 0.02 | 0.01 |
| Glycerophospholipid metabolism | 0.58 | 0.03 | 0.62 | 0.04 | 7.19E-04 | 0.03 | -0.04 |
| Glycine, serine and threonine metabolism | 1.14 | 0.03 | 1.07 | 0.07 | 4.74E-04 | 0.03 | 0.07 |
| RNA polymerase | 1.31 | 0.08 | 1.11 | 0.15 | 1.13E-04 | 9.46E-03 | 0.19 |
| Histidine metabolism | 1.41 | 0.08 | 1.23 | 0.18 | 1.15E-03 | 0.03 | 0.18 |
| Cysteine and methionine metabolism | 1.46 | 0.04 | 1.35 | 0.11 | 8.56E-04 | 0.03 | 0.11 |
| Terpenoid backbone biosynthesis | 1.49 | 0.05 | 1.41 | 0.08 | 9.03E-04 | 0.03 | 0.08 |
| Alanine, aspartate and glutamate metabolism | 1.82 | 0.05 | 1.74 | 0.08 | 1.83E-03 | 0.03 | 0.08 |
| Peptidoglycan biosynthesis | 2.13 | 0.05 | 2.04 | 0.09 | 1.41E-03 | 0.03 | 0.09 |

| **KEGG Pathways** | **HD-D28** | | **HD-D35** | | ***P*-values** | ***P*-values (corrected)** | **Difference between means** |
| --- | --- | --- | --- | --- | --- | --- | --- |
|  | **Mean (%)** | **SD (%)** | **Mean (%)** | **SD (%)** |  |  |  |
| Drug metabolism - other enzymes | 1.20 | 0.56 | 0.08 | 0.33 | 2.81E-07 | 4.70E-05 | 1.11 |
| Valine, leucine and isoleucine degradation | 0.45 | 0.07 | 0.33 | 0.06 | 8.07E-06 | 6.74E-04 | 0.12 |
| Glycine, serine and threonine metabolism | 1.16 | 0.05 | 1.07 | 0.07 | 4.66E-05 | 1.95E-03 | 0.10 |
| Carbon fixation pathways in prokaryotes | 1.20 | 0.07 | 1.06 | 0.10 | 4.66E-05 | 2.59E-03 | 0.14 |
| Phosphotransferase system (PTS) | 0.38 | 0.14 | 0.72 | 0.29 | 2.65E-04 | 8.86E-03 | -0.34 |
| Fructose and mannose metabolism | 0.98 | 0.11 | 1.17 | 0.17 | 5.00E-04 | 0.01 | -0.20 |
| Amino sugar and nucleotide sugar metabolism | 1.08 | 0.06 | 1.21 | 0.11 | 4.83E-04 | 0.01 | -0.13 |
| Galactose metabolism | 0.92 | 0.09 | 1.14 | 0.20 | 6.62E-04 | 0.01 | -0.22 |
| Starch and sucrose metabolism | 0.95 | 0.07 | 1.09 | 0.14 | 1.38E-03 | 0.03 | -0.14 |
| Protein processing in endoplasmic reticulum | 0.06 | 0.00 | 0.05 | 0.01 | 1.94E-03 | 0.03 | 0.01 |
| Citrate cycle (TCA cycle) | 1.01 | 0.11 | 0.86 | 0.15 | 2.15E-03 | 0.03 | 0.16 |
| Glycerophospholipid metabolism | 0.58 | 0.04 | 0.62 | 0.04 | 3.41E-03 | 0.04 | -0.04 |
| Neomycin, kanamycin and gentamicin biosynthesis | 0.02 | 0.08 | 0.15 | 0.15 | 3.38E-03 | 0.05 | -0.13 |
